# Genetic insights into the risk of hip osteoarthritis on stroke: A single-variable and multivariable Mendelian randomization

**DOI:** 10.1371/journal.pone.0313032

**Published:** 2025-01-09

**Authors:** Zhengze Zhang, Yanan Lian, Yuewen He, Hao Liu, Kai Meng, Yong Wang, Wuhua Ma

**Affiliations:** 1 The First Clinical Medical School of Guangzhou University of Guangzhou University of Chinese Medicine, Guangzhou, Guangdong, PR China; 2 Department of Anesthesiology, The First Affiliated Hospital of Guangzhou University of Chinese Medicine, Guangzhou, Guangdong, PR China; 3 Shandong Mental Health Center, Jinan, Shandong, PR China; 4 Department of Orthopaedics, Affiliated Hospital of Shandong University of Traditional Chinese Medicine, Jinan, Shandong, PR China; University College London, UNITED KINGDOM OF GREAT BRITAIN AND NORTHERN IRELAND

## Abstract

**Background:**

Hip osteoarthritis has been identified as a potential risk factor for stroke, with previous studies have demonstrated an association between hip osteoarthritis and stroke. This study aims to further elucidate the causal relationship between the two, employing Two-Sample and Multivariable Mendelian randomization methods.

**Methods:**

SNPs, derived from two extensive GWAS, served as instruments in exploring the association between genetically predicted hip osteoarthritis and stroke risk, utilizing two-sample Mendelian randomization. In Multivariable Mendelian randomization, factors such as cigarettes per day, alcoholic drinks per week, hypertension, body mass index, type 2 diabetes, C-reactive protein, rheumatoid arthritis were incorporated to further account for the independent causal effects of multiple correlated exposures.

**Results:**

Two-sample Mendelian randomization analysis revealed that hip osteoarthritis exerts a potential causal effect on any stroke, any ischemic stroke, and cardioembolic stroke, while it did not influence large artery stroke and small vessel stroke. Multivariable MR analysis indicated that the causal effect of hip osteoarthritis on any ischemic stroke and cardioembolic stroke was no longer evident after adjusting for C-reactive protein, and similarly, the effect on any ischemic stroke was not observed after adjusting for type 2 diabetes. However, the effects on any stroke, any ischemic stroke, and cardioembolic stroke remained significant after adjustments for hypertension, alcoholic drinks per week, cigarettes per day, body mass index, and rheumatoid arthritis.

**Conclusion:**

The study demonstrated that elevated hip osteoarthritis, as predicted by genetic factors, was potential associated with an increased risk of any stroke, any ischemic stroke, and cardioembolic stroke, but showed no correlation with hypertension, alcoholic drinks per week, cigarettes per day, type 2 diabetes, C-reactive protein, body mass index levels, and rheumatoid arthritis.

## Introduction

Hip osteoarthritis (HOA), is globally recognized as the most prevalent source of hip pain and functional impairment [1]. It represents a comprehensive pathology of the entire joint, including cartilage, bone and synovium [2]. Its pathology entails a diminution of the joint interspace, degradation of the articular cartilage, osteosclerosis within the subchondral bone, formation of osteophytes, and persistent inflammation [1]. The occurrence of HOA is on the rise, largely due to an aging population. Most patients with HOA suffer from continuous pain, decreased mobility, and a reduced quality of life [3]. This leads to healthcare expenses exceeding €400 billion annually in European countries [4], creating a substantial social and financial burden for both patients and society. Nevertheless, the exploration of the etiology and therapeutic for HOA has not progressed as rapidly as it has for knee Osteoarthritis (KOA). This divergence may stem from the more prevalent incidence of KOA. Hitherto, clinical investigations has primarily concentrated on KOA or a combination of hip and knee OA, with the inferences from these studies typically being extended to apply to HOA. Hip and knee OA show notable differences in areas like incidence rates, clinical manifestations, anatomical physiology, therapeutic interventions, and clinical governance [5]. Therefore, further research on HOA is required to enhance and update the currently available speculative evidence.

Stroke, encompassing both ischemic stroke (IS) and hemorrhagic stroke, is characterized by neurological impairment as a result of acute, localized damage of the central nervous system induced by vascular issues. Potentially modifiable risk factors for stroke encompass hypertension, tobacco usage, diabetes, hyperlipidemia, sedentary lifestyle, and atrial fibrillation [6]. The Global Burden of Disease Stroke Collaborators report that stroke is the second leading cause of death worldwide [7]. In 2019, there were 12.2 million cases of stroke worldwide, resulting in 6.55 million deaths. Presently, the emphasis on stroke prevention is acknowledged as a pivotal strategy, with projections indicating that approximately 85% of all strokes might be avertible [8]. Especially noteworthy are the alterable risk elements like tobacco consumption, and total cholesterol. These factors are gaining increasing attention in stroke prevention efforts, as the incidence of stroke has declined by about 42% in developed countries during the past three decades [9]. Consequently, the identification and modification of risk elements could contribute to a decrease in the incidence of stroke.

Recent observational reports have indicated HOA is potentially a risk factor for stroke. A study involving 221,807 individuals from the United Kingdom reported an association between HOA and an elevated risk of stroke [10]. A 2020 large-scale cohort study with more than 320,000 participants confirmed a positive correlation, showing that HOA was linked to a 1.20 times higher risk of stroke [11]. A recently published systematic review and meta-analysis, which encompassed 42 observational studies, found patients diagnosed with OA were more prone to having various chronic diseases. Among these, stroke was identified as the most prominent co-morbidity linked to OA [12]. Recent studies have indicated the thickness of the carotid intima-media and carotid plaque are increased in patients with OA [13]. Nonetheless, two distinct meta-analyses, which underwent statistical adjustments to account for potential confounding factors such as study design (prospective vs. retrospective), diagnostic criteria for osteoarthritis age, sex, smoking, alcoholic intake, and body mass index, both arrived at the consensus that there is no increased risk of stroke for individuals with osteoarthritis [14, 15].

Since OA and stroke are frequently diagnosed as co-existing diseases, epidemiological studies have progressively explored the relationship between these prevalent ailments. The disparities observed in observational studies may stem from factors such as study design limitations, restricted sample sizes that are insufficient to address endogeneity, and biases resulting from residual confounding, such as the influence of OA medications and reverse causality. These biases may hinder the production of impartial causal estimates. Furthermore, OA and stroke share certain risk factors, such as obesity, diabetes, atherosclerosis, and metabolic syndrome [16]. This could impede the recognition of the link between HOA and Stroke or result in its underestimation.

To address the discrepancies in observational studies, this study opts for Mendelian randomization (MR), an epidemiological method that employs genetic variants, namely single-nucleotide polymorphisms (SNPs), as instrumental variables (IVs) to investigate the causal relationship between exposures and outcomes [17]. The SNPs, closely associated to the exposures and outcomes, undergo random arrangement at conception, making MR to be natural randomized controlled trial and avoiding reverse causation or confounding factors, and it is appropriate to investigate the association between HOA and Stroke while enabling for causal inference. Furthermore, Multivariable Mendelian randomization (MVMR) is an enhancement that can provide causality estimation for multiple exposures on a single outcome. This is particularly advantageous when dealing with multiple related risk factors and accounts for the observed pleiotropy.

In this study, MVMR was employed to address endogeneities and provide causal estimation regarding the relationship existing between HOA and stroke, while adjusting for the influence of hypertension, alcoholic drinks per week, Cigarettes per Day, Type 2 diabetes (T2D),C-reactive protein (CRP), body mass index (BMI), and rheumatoid arthritis. While a previous study by Zhao et al. investigated the causal relationship between HOA and stroke using MR [18], this study distinguishes itself by utilizing the MVMR approach to assess the impact of confounders on the relationship between HOA and stroke, and aims to provide clarity on the inconsistent findings regarding the relationship between HOA and stroke.

## Methods

### 1. Study design

As the study was a re-analysis of data from published studies or publicly available databases, no additional ethical approval was required. Our investigation followed the principles described in the Strengthening the Reporting of Observational Studies in Epidemiology (STROBE) guidelines, especially applied to MR.

**Fig 1:**
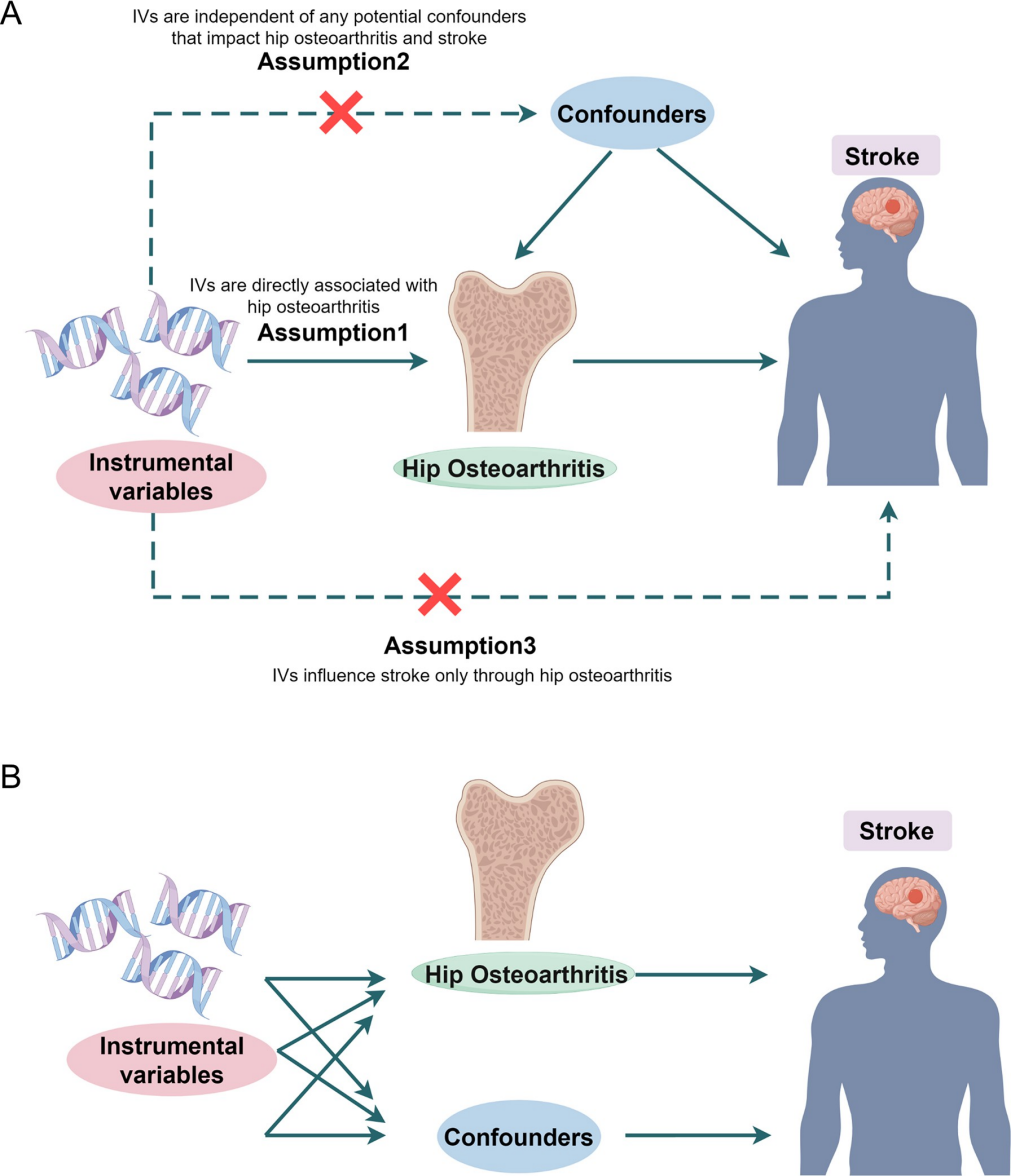
The flowchart of the study. Overview of the Two-Sample **(A)** and Multivariable Mendelian randomization **(B)** study design. Drawing by Figdraw.

The schematic representation of this study’s design is shown in Fig 1. In summary, the causative effects of HOA on stroke and its subtypes were initially estimated using Single variable Mendelian Randomization (SVMR). In order to negate the impact of confounding variables and to scrutinize the direct causative relationship between HOA and stroke, along with its subtypes, we conducted MVMR. It possesses the proficiency to ascertain the causative impact of various risk factors of stroke concurrently, thereby facilitating the identification of the independent link between each risk exposure and the resulting outcome [19].

Previous studies have widely recognized Hypertension, alcoholic drinks per week, Cigarettes per Day, T2D, CRP, BMI, and rheumatoid arthritis as modifiable risk factors for stroke [16, 20, 21]. In our study, we used MVMR to test whether exposures to such six risk elements influence the relationship between HOA and stroke. In the MVMR analyses, the SNPs used were those that overlapped in HOA and the aforementioned confounding factors.

### 2. Data source

#### Hip osteoarthritis

To determine the genetical associations with HOA, summary-level data were obtained by the largest genome-wide meta-analysis to date [22]. This analysis encompassed 826,690 participants, including 177,517 cases and 649,173 controls. The average age was 62.4 years (with a standard deviation of 11.9) for cases and 52.4 years (standard deviation of 17.4) for controls. The proportion of females was 62% in cases and 52% in controls. This meta-analysis combined data from 13 cohorts, predominantly (>97%) of European descent.

In our study, we utilized data from HOA cases (n = 36,445) and a maximum number of healthy controls (n = 333,557). HOA was defined based on criteria established by the GO, which included self-reported status, hospital diagnoses, International Classification of Diseases-10 (ICD-10) codes, or radiographic definitions provided by the TREAT-OA Consortium [23].

#### Stroke

Data on stroke and its subtypes were derived from summary level datasets from a meta-analysis by the MEGASTROKE consortium in the Europeans [24]. This dataset contained cases of any stroke (AS, n case = 40,585), any ischemic stroke (AIS, n case = 34,217), large artery stroke (LAS, n case = 4,373), cardioembolic stroke (CES, n case = 7,193), and small vessel stroke (SVS, n case = 5,386).

Stroke diagnosis was based on the criteria established by the World Health Organization (WHO). They identify it as the rapid development of signs indicating focal or global cerebral dysfunction, lasting for over 24 hours or progressing to death, with no identifiable cause other than vascular origin. Subsequent categorization of IS into LAS, CES, and SVS was implemented in accordance with the Trial of Org 10,172 in Acute Stroke Treatment (TOAST) criteria [25]. Further details regarding these study cohorts, precise methods of stroke identification, and subtyping are available in prior publications [24].

#### Other

The genetics information on cigarette and alcoholic beverage consumption was obtained from the GWAS and Sequencing Consortium of Alcohol and Nicotine use (GSCAN), comprising sample sizes of 335,394 for Cigarettes per Day and 337,334 for alcoholic drinks per week [26]. Data on BMI were obtained from genome-wide association studies and Metabochip meta-analyses conducted by Locke et al. [27]. The data for T2D and CRP were sourced from the GWAS meta-analyses conducted by Ligthart et al. [28] and Xue et al. [29]. The data pertaining to hypertension were compiled from the FinnGen biobank analysis round 9, including 111,581 patients and 265,626 controls [30]. The GWAS summary data on rheumatoid arthritis were retrieved from the Integrative Epidemiology Unit OpenGWAS database which obtained from 1,605 cases and 359,589 controls and covered 10,079,899 SNPs. This dataset was sourced using the keyword “Rheumatoid arthritis” and selected from the UK Biobank database results.

The comprehensive characteristics of all the GWAS datasets used in our investigation, encompassing the mediator information, are outlined in S1 Table. And we obtained access to the data through an online application process.

### 3. Genetic instrument selection

To ensure statistical rigor in selecting IVs, we performed a series of quality checks to satisfy the three core hypotheses for potential IVs. The conditionally independent IVs were chosen based on a genome-wide significance threshold of *p* < 5 × 10^−8^, with further quality control considering a minor allele frequency (MAF) greater than 1% to ensure the statistical efficacy of the IVs. We then assessed the linkage disequilibrium (LD) of the SNPs. SNPs with an r^2^ greater than 0.001 were excluded, using a window size of 10,000 kb based on the 1,000 Genomes European reference panel. The HLA was excluded from the study due to its intricate genetic architecture and significant linkage disequilibrium, which could potentially introduce bias into the analysis results.

To calculate *R2*, the proportion of variance in exposure that is explained by a particular genetic variation, we used the equation: *R*^*2*^ = 2 × β^2^× EAF × (1−EAF). In this equation, the symbol β denotes the estimated effect of the genetic variation, while EAF signifies the frequency of the effect allele. Then we calculated the F-statistic using the equation: F = *R*^*2*^× (*N*–*k*−1)/*k* (1−*R*^*2*^), where *N* is the number of samples and *k* is the number of genetic variants included [31]. Genetic variants with an F-statistic of less than 10 were regarded as weak IVs and removed from the MR analysis. Additionally, palindromic SNPs were removed from consideration. To ensure that genetic instruments were not associated with confounders, we used the PhenoScanner V2 to consider potential confounders [32].

### 4. Main statistical analysis

All analyses were performed in R software (version 4.3.0) using the R package “TwoSampleMR” [33]. Causal associations between HOA and risk of stroke were expressed using ORs with 95% CIs. It should be noted that since these estimates have no clear interpretation other than direction, they are only used to detect the presence of causal effects.

In our MR analysis, we utilized the inverse variance-weighted (IVW) method which provides the highest statistical power to estimate the causal link between exposures and outcomes [34]. Initially, it calculates the impact of each SNP on the outcome through the Wald ratio. Subsequently, the inverse variance of the SNPs serves as a weighting factor to amalgamate these influences into a unified causal estimate. The IVW with multiplicative random effects method provides a concise estimation and takes into account potential heterogeneity among the Wald ratio estimates from SNPs. Thus, if there is heterogeneity, random-effects IVW models (IVW-mre) are applied; otherwise, the fixed-effect IVW model (IVW-fe) is applied. This is due to the fact that IVW-fe models can sometimes produce artificially precise estimates [35, 36].

The MR-Egger, weighted median (WM), simple mode, and weighted mode were supplementary methods. Specifically, The WM method is a noteworthy technique, as it presents precise estimations despite 50% of the genetic variations not agreeing with essential MR assumptions [37]. On the contrary, the MR-Egger method is efficient in determining causality, irrespective of the absence of genetic variants that fulfill the core MR assumptions [38].

Robust significant causality indicates that at least one of the three main methods (IVW, MR-Egger, and WM) suggests a significant causal relationship and that the Beta values of the MR analyses of the three methods are in the same direction. Bonferroni’s multiple tests were used to correct for P value. A P value of less than 0.05 was considered suggestive of potential causality.

### 5. Sensitivity analysis

Furthermore, we employed Cochran’s Q statistics to assess heterogeneity in our analysis. P value less than 0.05 indicates the presence of heterogeneity [39]. To examine the possibility of directional pleiotropy, we used the MR-Egger method. In this context, directional pleiotropy refers to the scenario where the genetic variants used as IVs affect the outcome through pathways other than the exposure of interest. The existence of directional pleiotropy is suggested when P value for the intercept of the MR-Egger method are less than 0.05 [40].

Additionally, we conducted a "leave-one-out" sensitivity analysis [41]. This method involves systematically removing one SNP at a time from the analysis to see if any single SNP disproportionately influences the causal estimation. This approach helps to assess whether the discerned causal linkages between HOA and stroke are mediated through pivotal SNPs, thereby affirming the steadfastness of our causal evaluation.

### 6. Multivariable Mendelian randomization

To investigate the direct causal influence of HOA on stroke and its various subtypes, we undertook a MVMR study. This methodology is especially advantageous for concurrently discerning the causal impacts of multiple risk determinants on an outcome, like stroke, and for distinguishing the independent correlation of each risk elements with the outcome [19].

In our study, the potentially confounding factors considered for MVMR analysis comprised hypertension, alcoholic drinks per week, cigarettes per day, T2D, CRP, BMI, and rheumatoid arthritis. The SNPs used in the MVMR analyses were those that overlapped between HOA and these confounders. For the causal estimates in the multivariable analysis, we employed the IVW method. This method is highly effective for combining the effects of multiple genetic variants on an outcome, providing a robust estimate of the overall causal effect.

## Results

### 1. Screening of genetic instruments

A series of quality control checks were conducted to ensure the statistical validity of the three core assumptions for potential IVs [42]. SNPs were initially selected based on genome-wide significance (*p* < 5 × 10^−8^), yielding 3,080 SNPs. Linkage disequilibrium was then addressed by excluding SNPs with r^2^ > 0.001, using the 1000 Genomes European reference panel with a kb threshold of 10,000, resulting in 42 SNPs. After thorough screening for F-statistics, MAF < 0.01, and the exclusion of confounding factors via PhenoScanner, no SNPs were removed. Finally, after excluding palindromic sequences, 40 SNPs were identified as IVs for HOA (Table 1, S1 Table).

**Table 1:** Instrumental variables used in the two-sample MR analysis.

SNP	Effect allele	Other allele	beta	eaf	se	pval	F-STAT	Gene	Consequence
rs10465114	A	G	0.0625	0.2203	0.010874	9.04E-09	33.0371	RALGPS1	Intron Variant
rs1046934	A	C	0.0712	0.6493	0.009409	3.81E-14	57.2656	TSEN15	Missense Variant
rs10492367	T	G	0.1147	0.1983	0.01128	2.75E-24	103.3942	NA	NA
rs10831477	T	G	0.0704	0.8137	0.011579	1.20E-09	36.9643	MAML2	Intron Variant
rs10940168	A	G	-0.0534	0.3942	0.009248	7.74E-09	33.3399	LOC105379013	Intron Variant
rs11164653	T	C	-0.0799	0.4131	0.009162	2.77E-18	76.0472	COL11A1	Intron Variant
rs111844273	A	G	0.2317	0.0209	0.032524	1.05E-12	50.7499	HDAC9	Intron Variant
rs11727676	T	C	-0.0849	0.901	0.015484	4.18E-08	30.0658	HHIP	Synonymous Variant
rs12160491	A	G	-0.0614	0.7047	0.009839	4.37E-10	38.9408	NA	NA
rs12209223	A	C	0.1398	0.1117	0.014341	1.88E-22	95.0265	LOC101928540	Non Coding Transcript Variant
rs13302198	T	C	0.0544	0.6445	0.009918	4.14E-08	30.0826	DELEC1	Intron Variant
rs17677724	T	C	0.0723	0.1611	0.012245	3.54E-09	34.8641	LOC105379168	Intron Variant
rs1809889	T	C	0.0596	0.2806	0.010098	3.58E-09	34.8379	NA	NA
rs189933136	T	C	0.114	0.8413	0.01458	5.33E-15	61.1343	GSDMC	Intron Variant
rs1913707	A	G	0.0677	0.6043	0.009197	1.82E-13	54.1855	LOC124900668	Non Coding Transcript Variant
rs2188730	A	G	0.0722	0.1505	0.012602	1.01E-08	32.8217	NA	NA
rs2268023	A	T	0.0678	0.4109	0.009184	1.56E-13	54.4978	ITIH1	Intron Variant
rs2416564	T	C	-0.0734	0.5981	0.009145	1.00E-15	64.4262	ASTN2	Intron Variant
rs2521348	T	C	0.0555	0.3908	0.009192	1.56E-09	36.4577	MAP2K6	Intron Variant
rs2605098	A	G	0.0651	0.3368	0.009488	6.83E-12	47.0768	LYPLAL1-AS1	Intron Variant
rs28567725	T	C	-0.0546	0.5732	0.009066	1.72E-09	36.2674	FTO	Intron Variant
rs2862851	T	C	0.0655	0.4653	0.009022	3.86E-13	52.7134	TGFA	Intron Variant
rs3740129	A	G	0.0547	0.4505	0.009099	1.84E-09	36.1413	CHST3	Missense Variant
rs4073717	T	G	-0.0672	0.2013	0.011277	2.54E-09	35.5117	FGF18	Intron Variant
rs4252548	T	C	0.2254	0.0276	0.028433	2.24E-15	62.8449	IL11	Missense Variant
rs4411121	T	C	0.0648	0.3137	0.009679	2.16E-11	44.8189	NA	NA
rs62578126	T	C	-0.0626	0.3619	0.009494	4.29E-11	43.4762	LMX1B-DT	Intron Variant
rs66989638	A	G	0.0789	0.1326	0.013477	4.79E-09	34.2739	ECRG4	Intron Variant
rs6786146	T	C	0.1156	0.9479	0.020567	1.90E-08	31.5909	VGLL4	Intron Variant
rs67924081	A	G	0.0642	0.7441	0.010441	7.80E-10	37.8083	EHBP1L1	upstream transcript variant
rs6855246	A	G	-0.1087	0.9277	0.01884	7.94E-09	33.2896	NA	NA
rs6908606	A	G	-0.0688	0.711	0.00991	3.86E-12	48.1931	SUPT3H	Intron Variant
rs7875152	A	C	-0.0935	0.1408	0.013129	1.07E-12	50.7184	LOC105376205	Intron Variant
rs788857	A	G	-0.0544	0.7004	0.009939	4.42E-08	29.9574	PRKG2	Intron Variant
rs79056043	A	G	-0.1136	0.9367	0.018937	1.99E-09	35.9842	LRIG3	Intron Variant
rs79220007	T	C	-0.1059	0.9265	0.017705	2.22E-09	35.7745	HFE	3 Prime UTR Variant
rs798756	T	C	-0.0683	0.1936	0.011422	2.24E-09	35.7544	SLBP	Intron Variant
rs9475400	T	C	0.1084	0.0987	0.015138	8.03E-13	51.2748	BMP5	Intron Variant
rs9835230	A	G	0.0638	0.2433	0.010523	1.34E-09	36.7555	P3H2	Intron Variant
rs9976458	T	G	0.0628	0.2329	0.01125	2.37E-08	31.1624	ERG	Intron Variant

### 2. Causal effect estimates of HOA on AS, AIS, CES in two-sample Mendelian randomization

**Fig 2:**
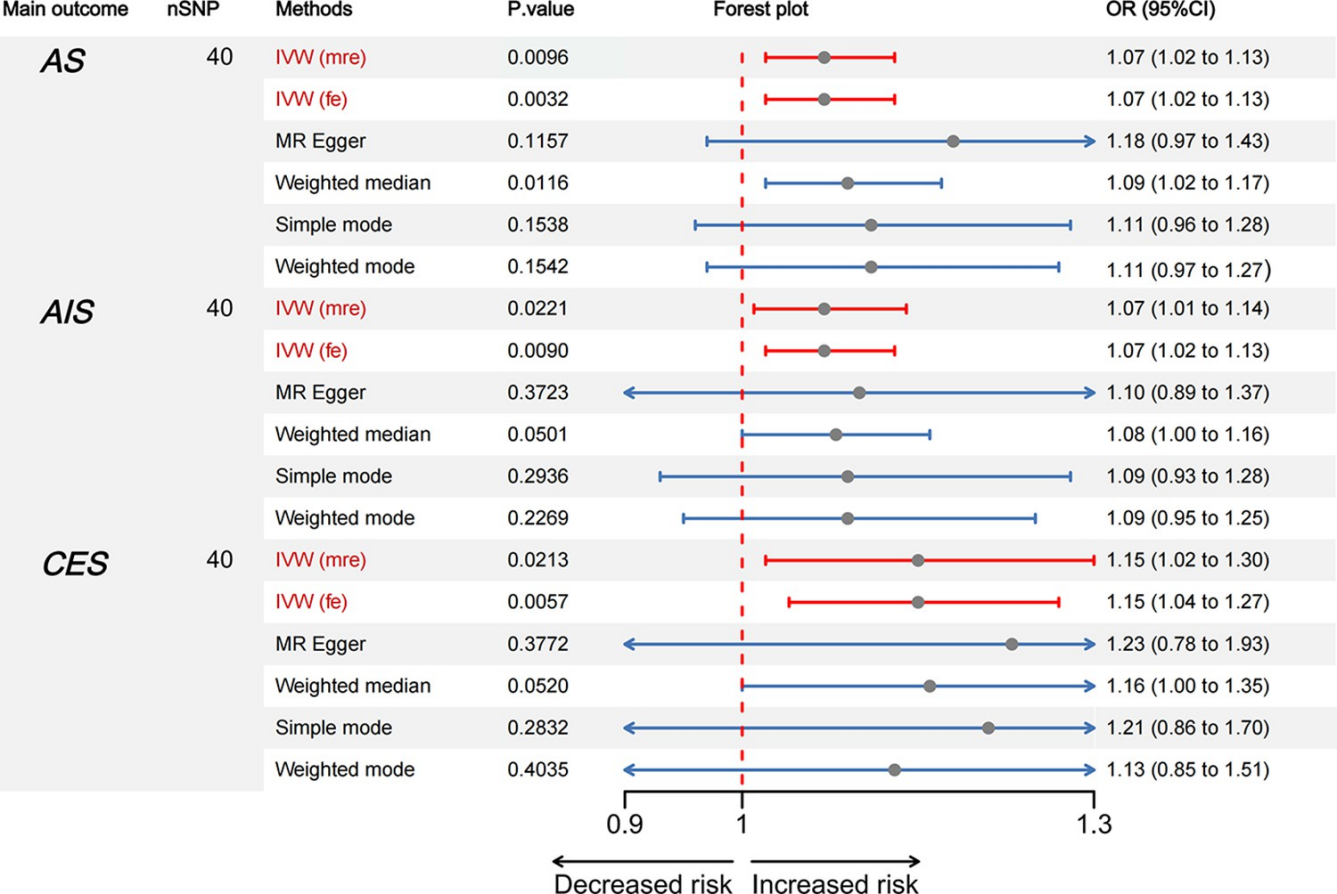
Forest plots showing the causal relationship between HOA and AS, AIS, CES. The causal estimates between exposure and outcome were expressed as odds ratio (OR) and 95% confidence interval (CI). nSNP: the number of SNP, IVW (mre): Inverse Variance Weighted multiple-random-effects model, IVW (fe): Inverse Variance Weighted fixed-effects model, AS: any stroke, AIS: any ischemic stroke, CES: cardioembolic stroke.

As depicted in Fig 2, our MR analysis indicated a statistically significant association between HOA and AS. The odds ratio observed suggests a possible association, though the effect size appears to be small. (*p* = 0.0096, OR = 1.07, 95% (CI) = 1.02–1.13 for the IVW multiplicative random effects (IVW-mre); *p* = 0.0032, OR = 1.07, 95% (CI) = 1.02–1.13 for the IVW- fixed effects method (IVW-fe).

**Fig 3:**
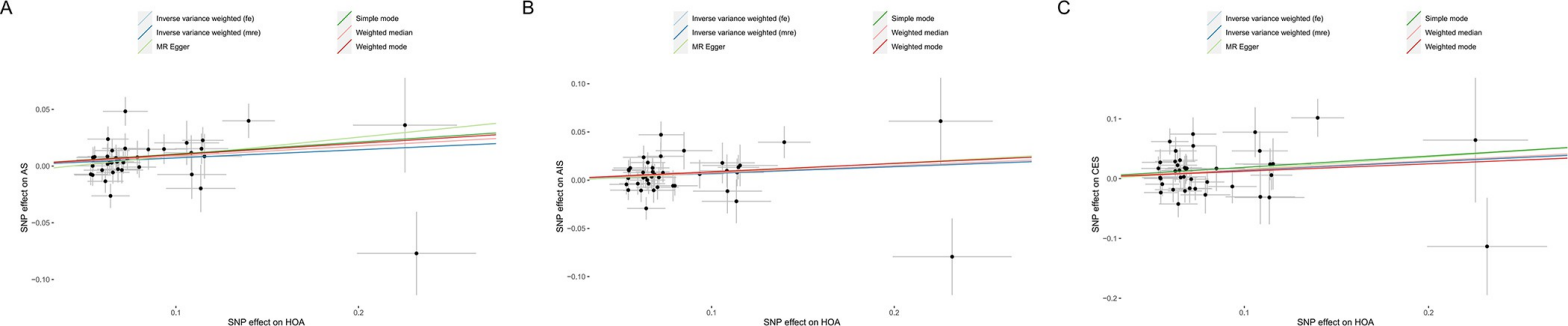
The scatter plot between HOA and AS **(A)**, AIS **(B)**, CES **(C)**.

**Fig 4:**
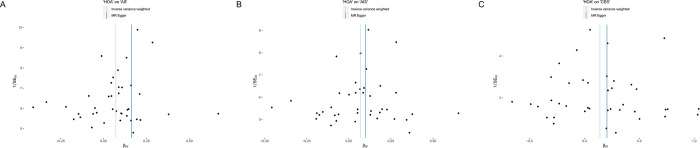
The funnel plot between HOA and AS **(A)**, AIS **(B)**, CES **(C)**.

**Fig 5:**
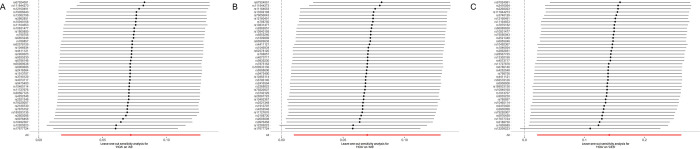
The leave-one-out analysis between HOA and AS **(A)**, AIS **(B)**, CES **(C)**.

The scatter plot showcased in Fig 3A demonstrates that with the intensification of the effect of IVs on HOA, there is a concurrent augmentation in the influence of SNPs on AS. Cochran’s Q test (Table 2) and funnel plot (Fig 4A) signified a homogeneity within the dataset. (Q = 50.67; *p* = 0.099). The intercept and the P value of the MR-Egger method were -0.007 and *p* = 0.358 (>0.05) respectively (Table 2), which statistically reinforces the inference that the IVs exhibited no horizontal pleiotropy. To assess the stability of our findings, we conducted leave-one-out sensitivity tests. The results of this analysis confirmed that the robustness of the MR results remained intact regardless of the removal of any single SNP, as depicted in Fig 5A.

**Table 2:** Sensitivity analyses of the causal association between HOA and stroke.

Exposure: Outcome	MR-Egger regression ^a^		Heterogeneity analyses Cochrane’s Q test ^b^	
	Intercept	Intercept_p	*Q*	*Q_pval*
AS	-0.007	0.358	50.67	0.099
AIS	-0.002	0.778	50.74	0.099
CES	-0.005	0.774	56.09	0.037
LAS	-0.006	0.789	52.66	0.071
SVS	-0.035	0.078	58.47	0.023

Q: Cochran’s Q statistics. The Cochrane’s Q test quantifies the effect of heterogeneity (*p* < 0.05, which means possible heterogeneity, thus prioritizing IVW(mre) methods). The MR-Egger intercept quantifies the effect of directional pleiotropy (*p* < 0.05, which means possible directional pleiotropy).

Similarly, the MR analysis provided statistical evidence indicating a potential causal effect of HOA on the development of both AIS and CES. This suggests that HOA may contribute to an increased likelihood of these conditions, highlighting a possible underlying genetic influence in their risk profiles.

HOA was found to elevate the potential risk of AIS, (*p* = 0.0221, OR = 1.07, 95% (CI) = 1.01–1.14 for the IVW-re estimator;*p* = 0.0090, OR = 1.07, 95% (CI) = 1.02–1.13 for the IVW-fe estimator.) (Fig 2). The scatter plot elucidates that the risk of AIS amplifies in tandem with the effect of IVs on HOA (Fig 3B). Both the Cochran’s Q (CQ) test and the funnel plot (Fig 4B) revealed no evidence of heterogeneity. (Q = 50.74; *p* = 0.099). The MR-Egger test presented an intercept term of -0.002, *p* = 0.778 (Table 2), suggesting the absence of pleiotropy. Furthermore, the sequential exclusion of each SNP demonstrated that no individual SNP exerted a significant effect in the results (Fig 5B).

Following a sensitivity analysis, the Cochran’s Q (Q = 56.09; *p* = 0.037;) test and funnel plot (Fig 4C) indicated heterogeneity in the findings (Table 2). If there is no excess heterogeneity in the tests, the IVW-mre and IVW-fe results will be identical, resulting in no loss of precision. However, if excess heterogeneity is present, the IVW-fe estimate may be overly precise, and this heterogeneity must be considered. If statistical evidence for a causal effect is found in IVW-mre analysis, it indicates that the genetic variants support a causal effect of the exposure on the outcome, even when accounting for heterogeneity. The outcomes of the IVW-mre analysis indicate that there may be an association between genetically predicted HOA and an increased risk of CES. (*p* = 0.0213, OR = 1.15, 95% (CI) = 1.02–1.3 for the IVW-re estimator; *p* = 0.0057, OR = 1.15, 95% (CI) = 1.04–1.27 for the IVW-fe estimator.) (Figs 2 and 3C). Featuring an intercept value of -0.005, and a P value of 0.774 (Table 2), the MR-Egger analysis did not reveal any evidence of pleiotropy. The leave-one-out test confirmed that the association with HOA and CES did not contingent upon a single SNP (Fig 5C).

### 3. Causal effect estimates of HOA on LAS, SVS in SVMR

S1 Fig demonstrates that HOA lacked a causal link with LAS or SVS, as their P value were above 0.05. The scatter plots depicting the influence of HOA on LAS or SVS showed no increase in their risk (S2 Fig). A disparity was noted in the heterogeneity between HOA and SVS, but not between HOA and LAS, as indicated by Cochran’s Q test results (Table 2, S3 Fig). No evidence of pleiotropy was found in either instance. The robustness of the leave-one-out analysis for LAS and SVS persisted, even when any single SNP was omitted (S4 Fig). The causal impacts of each IV on AS, AIS, CES, LAS, and SVS were illustrated in S5–S9 Figs.

### 4. Causal effect estimates of HOA on AS,AIS,CES in MVMR

**Fig 6:**
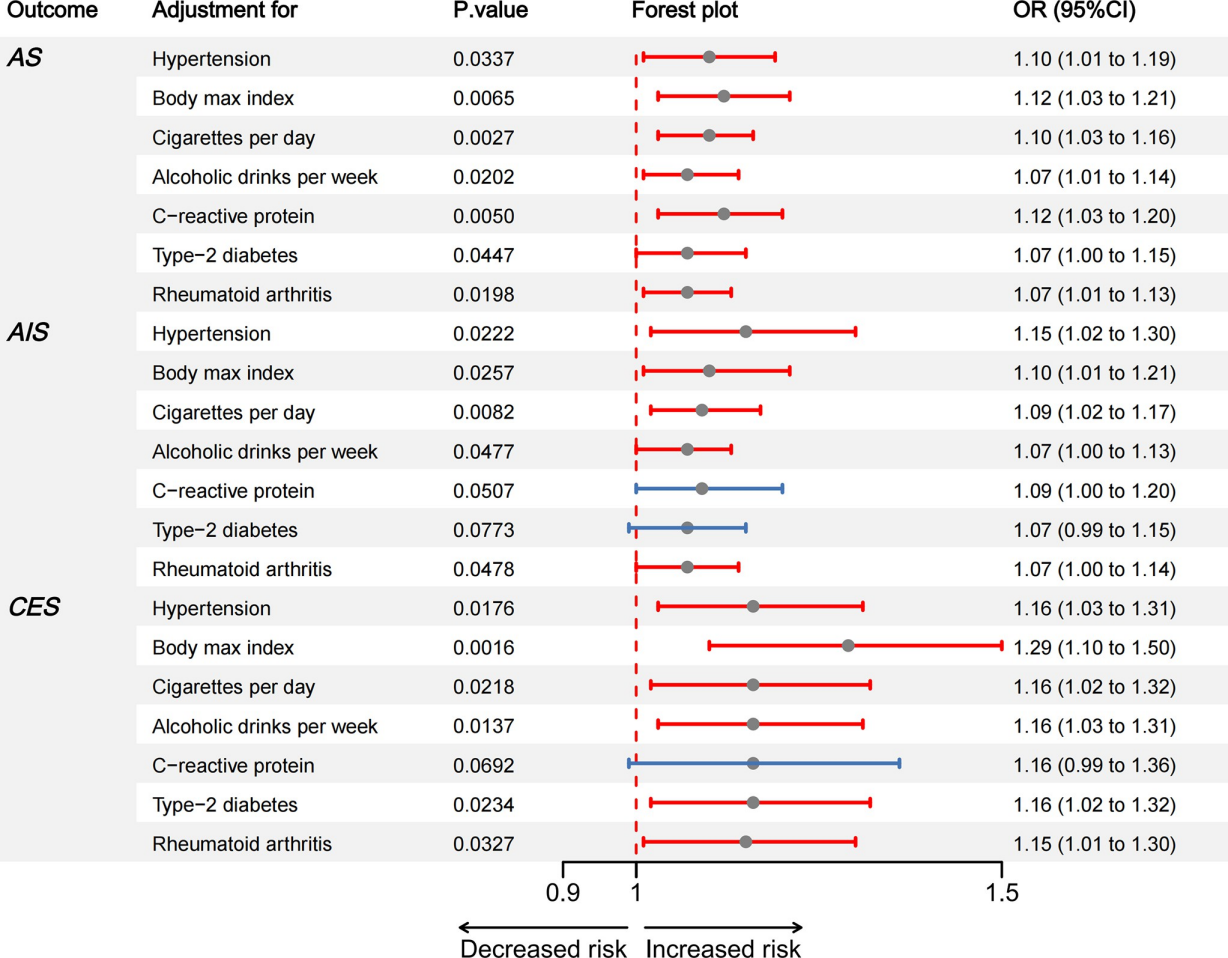
Causal estimates of HOA on AS, AIS, CES in Multivariable MR. Hypertension, alcoholic drinks per week, cigarettes per day, T2D, CRP, BMI and rheumatoid arthritis were adjusted in the Multivariable MR analyses.

To further mitigate the pleiotropy, a MVMR analysis was conducted to more comprehensively assess the causal impacts of HOA on Stroke and its subtypes. As depicted in Fig 6, the causal effects of HOA on AS, AIS, and CES remained consistent after adjustments were made for variables including Cigarettes per Day, Hypertension, Alcoholic Drinks per Week, BMI, and rheumatoid arthritis.

The causal impact of HOA on stroke persisted unaltered when adjusted for CRP levels in AS. (AS, *p* = 0.0050, OR = 1.12, 95% CI = 1.03–1.20). However, the previously observed positive association between HOA and Stroke vanished when considering the effects of HOA on AIS or CES. (AIS, *p* = 0.0507, OR = 1.09, 95% CI = 1.00–1.20)、(CES, *p* = 0.0692, OR = 1.16, 95% CI = 0.99–1.36) (Fig 6).

After accounting for T2D, a comparable scenario unfolded. The previously positive causal relationship between HOA and AIS dissipated(AIS, *p* = 0.0773, OR = 1.07, 95% CI = 0.99–1.15), while the impact of HOA on AS and CES continued to persist. (AS, *p* = 0.0447, OR = 1.07, 95% CI = 1.00–1.15)、(CES, *p* = 0.0234,OR = 1.16,95% CI = 1.02–1.32) (Fig 6).

### 5. Causal effect estimates of HOA on LAS and SVS in MVMR

Upon adjusting for confounding variables, no substantial causal relationship was identified for LAS or SVS, with all P value exceeding 0.05, as detailed in S10 Fig. Subsequently, the SVMR study disclosed an absence of causal effects of HOA on these stroke subtypes. Thus, our study provides insights indicating that HOA does not increase the incidence for these particular forms of stroke.

## Discussion

Based on the findings from GWAS summary data and SVMR analysis, our study suggests a potential causal relationship between HOA and the risks of AS, AIS, and CES. Although the p-value indicates statistical significance, the odds ratio, which is close to 1, suggests a minor effect size. Therefore, the strength of this association should be interpreted with caution, as it may reflect a weak or potential effect. And we do not observe a causal association between HOA and LAS or SVS. In order to avoid the possible confounding effects of SVMR, We conducted the MVMR study. It is demonstrated that increased HOA is associated with a causal increase in the risk of AS, AIS, CES, independent of Cigarettes per Day, alcoholic drinks per week, BMI, hypertension and rheumatoid arthritis. After controlling for CRP, HOA was still associated with AS. The effects of HOA on AS and CES remained unchanged after adjusting for T2D. Our study provides new, unbiased evidence of HOA’s role in preventing stroke, potentially aiding in reducing the high incidence of this condition.

After accounting for various related factors in a population cohort study of 720,055 British Columbians. Atiquzzaman et al. [43] discovered that individuals diagnosed with OA had a 14% increased incidence of stroke. However, 64% of OA impact on elevating the risk of stroke was attributed to the use of non-steroidal anti-inflammatory drugs (NSAIDs). When adjusted for potential covariates, the OA group demonstrated a 1.10-time higher incidence of stroke in comparison to the non-OA group [44]. According to the US NHANES III, OA is associated with an elevated incidence of metabolic syndrome [45]. Additionally, a recent meta-analysis investigating the correlation of OA and cardiovascular disease found no significant correlation between OA and stroke [14].

The discrepancies may be explained by residual confoundings, such as alcohol and cigarette use, which can be minimised by MR. The research encompassed in the meta-analysis exhibited diversity in their methodology, participant demographics, the extent of controlling for confounding elements, and diagnosis of both exposure and outcome. These variances probably lowered the reliability of their findings. Conducting a Randomized Controlled Trial (RCT) to explore the link between snoring and HOA is challenging in practice. However, our MR analysis offers new insights to address these confounding factors and reveals the causal association of HOA with stroke.

All 40 SNPs included in the analysis demonstrated a significant association with HOA and met the stringent criteria for IVs. These SNPs exhibited robust effect sizes and statistical significance in GWAS studies, making them suitable as IVs for subsequent analyses. For instance, COL11A1 (rs11164653) [46], FGF18 (rs4073717) [47], IL-11 (rs4252548) [48] and FTO (rs28567725) [49] were among the selected SNPs.

COL11A1 is involved in regulating protein digestion and absorption and is closely linked to the integrity of the blood-brain barrier during ischemic attack [50]. In an *in vivo* study, FGF18 was found to be effective in reducing infarct volume and ameliorating behavioral deficits in the stroke model. Additionally, FGF18 has been demonstrated to promote neurogenesis and stimulate neurite outgrowth [51]. The number of morphologically abnormal neurons significantly decreased following FGF18 treatment. The improved neuronal index correlated well with survival outcomes and aligned with previous findings showing a reduction in infarct size in rodents after FGF18 treatment [52]. In the pathological process of stroke, IL-11 can upregulate the expression of vascular cell adhesion molecule-1 (VCAM-1) through its proinflammatory effects, leading to vascular inflammation and injury. This upregulation contributes to the instability and rupture of atherosclerotic plaques, thereby increasing the stroke risk. Additionally, IL-11 may indirectly influence the expression and function of VCAM-1 by impairing vascular endothelial function and promoting thrombosis [53]. FTO plays a crucial role in regulating atherosclerosis by directly mediating the expression of CD40 ligand (CD40L). It binds to the m6A-specific sequence on CD40L mRNA, promoting its degradation, modulating the intensity of the immune response, and thereby reducing tissue damage [54]. The involvement of genes corresponding to these SNPs in stroke highlights their potential clinical utility. First, these genes may serve as biomarkers, allowing the identification of individuals at higher risk of stroke through the detection of specific SNP variations. Second, given the critical role these genes play in stroke pathology, they are promising candidates for therapeutic targets. Treatment plans can be tailored to the genetic characteristics of each patient, thereby enhancing treatment efficacy and prognosis. These findings offer new insights and directions for the prevention, diagnosis, and treatment of stroke, and future research may further explore the clinical applications of these genes.

The exact mechanisms underlying the association between hip OA and Stroke remain uncertain. However, possible explanations for this association might involve inflammatory factors, alterations in physical activity, and diabetes melitus. Additionally, the central mechanisms of secondary pain of hip OA could play a role.

Changes in physical activity among OA patients might also mediate this relationship, as physical inactivity is known to elevate stroke risk [55]. Intense articular pain during the OA period often prevents patients from participating in physical activity. This lack of activity is linked to the progression of hypertension, hyperlipidaemia, hyperglycaemia and an increased risk of diabetes mellitus. Moreover, physical inactivity can impair endothelial function and raise blood viscosity [56]. In addition to clinical studies, a genetic study of ischemic stroke revealed a common genetic overlap between physical activity and ischemic stroke [57], with the underlying mechanism involving exercise’s role in enhancing vascular and endothelial health.

Novel genetic loci associated with lacunar stroke were identified in a GWAS study involving 7338 stroke patients and 254798 controls. A multitrait approach was subsequently employed to detect additional genetic variants linked to lacunar stroke through a joint analysis of white matter hyperintensities from large-scale GWAS. The potential causal effect of diabetes on genetically-based lacunar stroke was also assessed, revealing a positive association [58].

On another note, the chronic pain which is the primary characteristic of OA patients impairs their physiological function and restricts physical movement. Chung et al. reported in a nation-wide population study that chronic pain in HOA could lead to an increased risk of stroke due to a stress response [59]. The stress response, often triggered by chronic pain, can affect the vasculature by raising blood pressure and reducing insulin sensitivity. This effect is mediated through the hypothalamic-pituitary-adrenal axis and the sympathetic nervous system [18, 59].

Additionally, responding to emotional stress, including pain, this axis system can release catecholamines, potentially leading to endothelial dysfunction. This dysfunction is a crucial early sign of atherosclerosis [60]. Notably, carotid atherosclerosis is recognized as a clinically impactful risk parameter for stroke [61]. A contemporary research identified heightened plasma concentrations of CD40L and VCAM-1 in women diagnosed with knee OA, in contrast to those devoid of the condition [13]. CD40L and VCAM-1 are integral contributors to the development of atherosclerosis. Hoeven et al. [62] reported that atherosclerosis, gauged through carotid intimal-medial thickness and the presence of carotid plaque, exhibits an association with OA. Osteoarthritis is distinguished by the degeneration of impacted joints and persistent low-grade inflammation.

Atherosclerosis, which can occur in both extracerebral and intracerebral arteries, is crucial in stroke pathogenesis. The link between inflammation and atherosclerosis is well-documented [63]. Systemic inflammation might indicate the mechanism that how OA can increase the risk of stroke. OA is known for the deterioration of affected joints and persistent low-grade inflammation. During OA progression, hypertrophic chondrocytes and proliferating synoviocytes secrete pro-inflammatory mediators as part of the repair mechanism of cartilage composition [64]. This persistent inflammation may trigger the renin-angiotensin system, potentially leading to oxidative stress. This, in turn, can result in endothelial dysfunction and atherosclerosis [65]. Total hip replacement (THR) is a mainstream therapeutic intervention for the symptomatic treatment of hip OA. This surgical technique may affect the activity of specific inflammatory cytokines and trigger inflammatory responses [66]. In a GWAS study of stroke and its subtypes, it has been confirmed that inflammatory factors, such as IL-1β, IL-12, and M-CSF, play a critical role in the onset of stroke and are closely associated with its occurrence [67].

There has been a MR Study that has also investigated the link between HOA to stroke. Their main findings were similar with ours. However, 7 pivotal factors, Cigarettes per Day, alcoholic drinks per week, BMI, hypertension, CRP, T2D and rheumatoid arthritis, were not adjusted in their analysis. A key advance is the application of the MVMR framework, which effectively addresses the endogeneity issues inherent in observational studies and accounts for related risk factors not considered in a single-variable MR. It is therefore hypothesized that HOA may have a direct contribution to the pathogenesis of stroke, rather than via the mediation of potentially modifiable risk factors. And the genetic data for OA utilized in our study were procured from the most comprehensive and recent genome-wide association study (GWAS) conducted to date by Boer’s group. and is the most comprehensive GWAS analysis available on osteoarthritis. Our analysis of stroke subtypes showed an association between HOA and AS, AIS, and CES, but no causal link was found between HOA and LAS or SVS. These observations suggest there may be biological heterogeneity in the genetic effects of HOA on various stroke subtypes., with each subtype potentially having its own unique pathological mechanisms.

This study has several limitations. One limitation is that the genetic variants were obtained from GWAS conducted in individuals of European ancestry. This ancestral focus may limit the broader applicability of our findings. To mitigate this, subsequent validation in diverse ancestral groups, such as Asian populations, is essential. And the findings offer an exploration of potential associations between exposure factors and outcomes. While statistically significant, the small effect size (with an OR close to 1) may constrain the ability to infer causality from these results. Consequently, these findings are better suited as a foundation for future research, where further validation is recommended to ascertain whether this association is indeed causal.

## Conclusion

After using single-variable and multi-variable MR, we found a causal relationship between HOA and AS, AIS, CES, and no association with other risk factors.

## Supporting information

S1 Table Summary of the GWAS datasets included in this MR study. (DOCX)

S2 Table Reports of SNPs across all phenotypes. (DOCX)

S1 Fig Forest plots showing the causal relationship between HOA and LAS, SVS. (TIF)

S2 Fig The scatter plot between HOA and LAS, SVS. (TIF)

S3 Fig The funnel plot between HOA and LAS, SVS. (TIF)

S4 Fig The leave-one-out analysis between HOA and LAS, SVS. (TIF)

S5 Fig The results of MR analyses of causal associations between each HOA SNP and AS. (TIF)

S6 Fig The results of MR analyses of causal associations between each HOA SNP and AIS. (TIF)

S7 Fig The results of MR analyses of causal associations between each HOA SNP and CES. (TIF)

S8 Fig The results of MR analyses of causal associations between each HOA SNP and LAS. (TIF)

S9 Fig The results of MR analyses of causal associations between each HOA SNP and SVS. (TIF)

S10 Fig Causal estimates of HOA on LAS, SVS in Multivariable MR. Hypertension, alcoholic drinks per week, cigarettes per day, T2D, CRP, BMI and rheumatoid arthritis were adjusted in the multivariate MR analyses.
(TIF)

S1 Raw data (ZIP)

## Abbreviations

HOA: Hip Osteoarthritis
KOA: Knee Osteoarthritis
IS: ischemic stroke
GWAS: Genome-Wide Association Study
MR: Mendelian Randomization
MVMR: Multivariable Mendelian Randomization
SVMR: : Single variable Mendelian Randomization
SNP: single-nucleotide polymorphisms
IV: instrumental variable
T2D: Type 2 diabetes
CRP: C-reactive protein
BMI: body mass index
AS: Any Stroke
AIS: Any Ischemic Stroke
LAS: Large Artery Stroke
CES: Cardioembolic Stroke
SVS: Small Vessel Stroke
WHO: World Health Organization
IVW: Inverse Variance Weighted
WM: Weighted Median
