# Supplementary material for: Genetic insights into the risk of hip osteoarthritis on stroke: A single-variable and multivariable Mendelian randomization
Source: PLoS One. 2025 Jan 9;20(1):e0313032. doi: 10.1371/journal.pone.0313032 (PMC11717317; doi:10.1371/journal.pone.0313032)
Supplement: S1 Table — (DOCX) [file pone.0313032.s001.docx]

| Phenotype | Consortium/Author | Year | N cases | N controls | N total | SNPs(N) | PMID | Open GWAS ID | link |
| --- | --- | --- | --- | --- | --- | --- | --- | --- | --- |
| Hip OA | Genetics of osteoarthritis consortium | 2021 | 36,445 | 316,943 | 353388 | 18,871,781 | 34450027 | NA | https://msk.hugeamp.org/downloads.html |
| All cause stroke | MEGASTROKE | 2018 | 40585 | 406111 | 446696 | 8211693 | 29531354 | ebi-a-GCST006906 | http://www.megastroke.org/download.html |
| Any ischemic stroke | MEGASTROKE | 2018 | 34217 | 406111 | 440328 | 8296492 | 29531354 | ebi-a-GCST006908 | http://www.megastroke.org/download.html |
| Cardioembolic stroke | MEGASTROKE | 2018 | 7193 | 204570 | 211763 | 8271294 | 29531354 | ebi-a-GCST006910 | http://www.megastroke.org/download.html |
| Large artery stroke | MEGASTROKE | 2018 | 4373 | 143572 | 150765 | 8418349 | 29531354 | ebi-a-GCST006907 | http://www.megastroke.org/download.html |
| Small vessel stroke | MEGASTROKE | 2018 | 5386 | 192662 | 198048 | 8280845 | 29531354 | ebi-a-GCST006909 | http://www.megastroke.org/download.html |
| Type-2 diabetes | Xue et. al | 2018 | 62892 | 596424 | 655666 | 5030727 | 30054458 | ebi-a-GCST006867 | http://www.megastroke.org/download.html |
| C-reactive protein | Ligthart et. al | 2018 | NA | NA | 204402 | 2414379 | 30388399 | ieu-b-35 | https://gwas.mrcieu.ac.uk/datasets/ieu-b-35/ |
| Body max index | Locke et. al | 2015 | NA | NA | 171977 | 2494613 | 25673413 | ieu-a-974 | https://gwas.mrcieu.ac.uk/datasets/ieu-a-974/ |
| Hypertension | Kurki et. al | 2023 | 111581 | 265626 | 377207 | 20175454 | 36653562 | finngen_R9_I9_HYPTENS | https://storage.googleapis.com/finngen-public-data-r9/summary_stats/finngen_R9_I9_HYPTENS.gz |
| alcoholic drinks per week | liu et. al | 2019 | NA | NA | 335394 | 11887865 | 30643251 | ieu-b-73 | https://gwas.mrcieu.ac.uk/datasets/ieu-b-73/ |
| Cigarettes per Day | liu et. al | 2019 | NA | NA | 337334 | 11913712 | 30643251 | ieu-b-25 | https://gwas.mrcieu.ac.uk/datasets/ieu-b-25/ |
| Rheumatoid arthritis | Neale lab | 2018 | 1605 | 359589 | 361194 | 10079899 | NA | ukb-d-M13_RHEUMA | https://gwas.mrcieu.ac.uk/datasets/ukb-d-M13_RHEUMA/ |

**Supplementary Table.1** Summary of the GWAS datasets included in this MR study.
