# Supplementary material for: Genetic insights into the risk of hip osteoarthritis on stroke: A single-variable and multivariable Mendelian randomization
Source: PLoS One. 2025 Jan 9;20(1):e0313032. doi: 10.1371/journal.pone.0313032 (PMC11717317; doi:10.1371/journal.pone.0313032)
Supplement: S2 Table — (DOCX) [file pone.0313032.s002.docx]

| Query | GWAS Trait | PMID | Position (GRCh37) | Alleles |
| --- | --- | --- | --- | --- |
| rs10465114 | Osteoarthritis (hip) | 34450027 | chr9:129917824 | A=0.218, G=0.782 |
| rs1046934 | Height | 20881960 | chr1:184023529 | A=0.673, C=0.327 |
| rs1046934 | Waist circumference adjusted for body mass index | 28448500 | chr1:184023529 | A=0.673, C=0.327 |
| rs1046934 | Waist circumference adjusted for body mass index | 28448500 | chr1:184023529 | A=0.673, C=0.327 |
| rs1046934 | Hip circumference adjusted for BMI | 25673412 | chr1:184023529 | A=0.673, C=0.327 |
| rs1046934 | Hip circumference adjusted for BMI | 25673412 | chr1:184023529 | A=0.673, C=0.327 |
| rs1046934 | Height | 31217584 | chr1:184023529 | A=0.673, C=0.327 |
| rs1046934 | Waist circumference adjusted for BMI (joint analysis main effects and physical activity interaction) | 28448500 | chr1:184023529 | A=0.673, C=0.327 |
| rs1046934 | Waist circumference adjusted for BMI in active individuals | 28448500 | chr1:184023529 | A=0.673, C=0.327 |
| rs1046934 | Waist circumference adjusted for BMI in active individuals | 28448500 | chr1:184023529 | A=0.673, C=0.327 |
| rs1046934 | Waist circumference adjusted for BMI in active individuals | 28448500 | chr1:184023529 | A=0.673, C=0.327 |
| rs1046934 | Waist circumference adjusted for BMI in inactive individuals | 28448500 | chr1:184023529 | A=0.673, C=0.327 |
| rs1046934 | Waist circumference adjusted for BMI (joint analysis main effects and physical activity interaction) | 28448500 | chr1:184023529 | A=0.673, C=0.327 |
| rs1046934 | Waist circumference adjusted for BMI (joint analysis main effects and physical activity interaction) | 28448500 | chr1:184023529 | A=0.673, C=0.327 |
| rs1046934 | Height | 36224396 | chr1:184023529 | A=0.673, C=0.327 |
| rs1046934 | Height | 36224396 | chr1:184023529 | A=0.673, C=0.327 |
| rs1046934 | Waist circumference adjusted for body mass index | 34021172 | chr1:184023529 | A=0.673, C=0.327 |
| rs1046934 | FVC | 36641522 | chr1:184023529 | A=0.673, C=0.327 |
| rs1046934 | Height | 36224396 | chr1:184023529 | A=0.673, C=0.327 |
| rs1046934 | Height | 36224396 | chr1:184023529 | A=0.673, C=0.327 |
| rs10492367 | Osteoarthritis (hip) | 30374069 | chr12:28014970 | G=0.796, T=0.204 |
| rs10492367 | Osteoarthritis of the hip or knee | 30664745 | chr12:28014970 | G=0.796, T=0.204 |
| rs10492367 | Osteoarthritis (hip) | 30664745 | chr12:28014970 | G=0.796, T=0.204 |
| rs10492367 | Surgical hip osteoarthritis | 36376028 | chr12:28014970 | G=0.796, T=0.204 |
| rs10492367 | Height | 36224396 | chr12:28014970 | G=0.796, T=0.204 |
| rs10492367 | Height | 36224396 | chr12:28014970 | G=0.796, T=0.204 |
| rs11164653 | Osteoarthritis (hip) | 34450027 | chr1:103464210 | C=0.576, T=0.424 |
| rs111844273 | Osteoarthritis (hip) | 34450027 | chr7:18436337 | A=0.026, G=0.974 |
| rs11727676 | Waist circumference adjusted for body mass index | 31669095 | chr4:145659064 | C=0.077, T=0.923 |
| rs11727676 | Body mass index | 25673413 | chr4:145659064 | C=0.077, T=0.923 |
| rs11727676 | Body mass index | 25673413 | chr4:145659064 | C=0.077, T=0.923 |
| rs11727676 | Brain region volumes | 31676860 | chr4:145659064 | C=0.077, T=0.923 |
| rs11727676 | High density lipoprotein cholesterol levels | 29507422 | chr4:145659064 | C=0.077, T=0.923 |
| rs11727676 | Body mass index | 25673413 | chr4:145659064 | C=0.077, T=0.923 |
| rs11727676 | High density lipoprotein cholesterol levels | 29507422 | chr4:145659064 | C=0.077, T=0.923 |
| rs11727676 | Body mass index | 26426971 | chr4:145659064 | C=0.077, T=0.923 |
| rs11727676 | Diverticular disease | 30177863 | chr4:145659064 | C=0.077, T=0.923 |
| rs11727676 | aparc-pial rh area pericalcarine | 33875891 | chr4:145659064 | C=0.077, T=0.923 |
| rs11727676 | aparc-DKTatlas lh area pericalcarine | 33875891 | chr4:145659064 | C=0.077, T=0.923 |
| rs11727676 | BA-exvivo lh area V1 | 33875891 | chr4:145659064 | C=0.077, T=0.923 |
| rs11727676 | BA-exvivo rh area V1 | 33875891 | chr4:145659064 | C=0.077, T=0.923 |
| rs11727676 | BA-exvivo rh area V2 | 33875891 | chr4:145659064 | C=0.077, T=0.923 |
| rs11727676 | aparc-a2009s rh area S-calcarine | 33875891 | chr4:145659064 | C=0.077, T=0.923 |
| rs11727676 | aparc-a2009s rh area G-cuneus | 33875891 | chr4:145659064 | C=0.077, T=0.923 |
| rs11727676 | IDP T1 FAST ROIs R intracalc cortex | 33875891 | chr4:145659064 | C=0.077, T=0.923 |
| rs11727676 | aparc-Desikan lh volume cuneus | 33875891 | chr4:145659064 | C=0.077, T=0.923 |
| rs11727676 | BA-exvivo lh volume V2 | 33875891 | chr4:145659064 | C=0.077, T=0.923 |
| rs11727676 | aparc-Desikan lh area pericalcarine | 33875891 | chr4:145659064 | C=0.077, T=0.923 |
| rs11727676 | aparc-pial lh area pericalcarine | 33875891 | chr4:145659064 | C=0.077, T=0.923 |
| rs11727676 | aparc-Desikan rh area lingual | 33875891 | chr4:145659064 | C=0.077, T=0.923 |
| rs11727676 | aparc-Desikan rh area pericalcarine | 33875891 | chr4:145659064 | C=0.077, T=0.923 |
| rs11727676 | aparc-pial rh area lingual | 33875891 | chr4:145659064 | C=0.077, T=0.923 |
| rs11727676 | aparc-a2009s lh area S-calcarine | 33875891 | chr4:145659064 | C=0.077, T=0.923 |
| rs11727676 | aparc-DKTatlas rh area lingual | 33875891 | chr4:145659064 | C=0.077, T=0.923 |
| rs11727676 | aparc-DKTatlas rh area pericalcarine | 33875891 | chr4:145659064 | C=0.077, T=0.923 |
| rs11727676 | Brain shape (segment 5) | 33821002 | chr4:145659064 | C=0.077, T=0.923 |
| rs11727676 | Serum levels of protein HHIP | 35078996 | chr4:145659064 | C=0.077, T=0.923 |
| rs11727676 | Waist-hip ratio | 30239722 | chr4:145659064 | C=0.077, T=0.923 |
| rs11727676 | Waist-to-hip ratio adjusted for BMI | 30239722 | chr4:145659064 | C=0.077, T=0.923 |
| rs11727676 | Colorectal cancer or advanced adenoma | 30510241 | chr4:145659064 | C=0.077, T=0.923 |
| rs11727676 | Waist circumference adjusted for body mass index | 34021172 | chr4:145659064 | C=0.077, T=0.923 |
| rs11727676 | Waist-to-hip ratio adjusted for BMI | 31669095 | chr4:145659064 | C=0.077, T=0.923 |
| rs11727676 | Body mass index | 30108127 | chr4:145659064 | C=0.077, T=0.923 |
| rs11727676 | Brain morphology (MOSTest) | 32665545 | chr4:145659064 | C=0.077, T=0.923 |
| rs11727676 | Nonalcoholic fatty liver disease (imputed) | 35047847 | chr4:145659064 | C=0.077, T=0.923 |
| rs11727676 | Alanine aminotransferase levels | 34594039 | chr4:145659064 | C=0.077, T=0.923 |
| rs11727676 | Fasting insulin | 34059833 | chr4:145659064 | C=0.077, T=0.923 |
| rs11727676 | Waist-to-hip ratio adjusted for BMI | 34021172 | chr4:145659064 | C=0.077, T=0.923 |
| rs11727676 | A body shape index | 34021172 | chr4:145659064 | C=0.077, T=0.923 |
| rs11727676 | Waist-hip index | 34021172 | chr4:145659064 | C=0.077, T=0.923 |
| rs11727676 | Body size at age 10 | 32376654 | chr4:145659064 | C=0.077, T=0.923 |
| rs11727676 | Cortical surface area | 34560273 | chr4:145659064 | C=0.077, T=0.923 |
| rs11727676 | Cortical thickness | 34560273 | chr4:145659064 | C=0.077, T=0.923 |
| rs11727676 | Vertex-wise sulcal depth | 34910505 | chr4:145659064 | C=0.077, T=0.923 |
| rs11727676 | Waist circumference adjusted for body mass index | 34021172 | chr4:145659064 | C=0.077, T=0.923 |
| rs11727676 | Vertex-wise cortical thickness | 34910505 | chr4:145659064 | C=0.077, T=0.923 |
| rs11727676 | Vertex-wise cortical surface area | 34910505 | chr4:145659064 | C=0.077, T=0.923 |
| rs11727676 | Colorectal cancer | 36539618 | chr4:145659064 | C=0.077, T=0.923 |
| rs11727676 | Brain morphology (MOSTest) | 35164939 | chr4:145659064 | C=0.077, T=0.923 |
| rs11727676 | Body surface area | 36502284 | chr4:145659064 | C=0.077, T=0.923 |
| rs11727676 | Body mass index (MTAG) | 36376304 | chr4:145659064 | C=0.077, T=0.923 |
| rs11727676 | Body mass index | 36581621 | chr4:145659064 | C=0.077, T=0.923 |
| rs11727676 | Body mass index or hip osteoarthritis (pleiotropy) | 36889626 | chr4:145659064 | C=0.077, T=0.923 |
| rs11727676 | Multi-trait sex score | 37277458 | chr4:145659064 | C=0.077, T=0.923 |
| rs11727676 | Lung function (forced vital capacity) | 36914875 | chr4:145659064 | C=0.077, T=0.923 |
| rs11727676 | Hedgehog-interacting protein levels | 34648354 | chr4:145659064 | C=0.077, T=0.923 |
| rs11727676 | Diverticular disease | 37492107 | chr4:145659064 | C=0.077, T=0.923 |
| rs11727676 | Alanine aminotransferase levels | 38632349 | chr4:145659064 | C=0.077, T=0.923 |
| rs11727676 | T1 brain MRIs Unsupervised Deep learning derived Imaging Phenotypes (dimension 32) | 38580839 | chr4:145659064 | C=0.077, T=0.923 |
| rs12160491 | Osteoarthritis (with total hip replacement) | 34450027 | chr22:38195796 | A=0.672, G=0.328 |
| rs12160491 | Smoking initiation | 36477530 | chr22:38195796 | A=0.672, G=0.328 |
| rs12209223 | Height | 25282103 | chr6:76164589 | A=0.11, C=0.89 |
| rs12209223 | Osteoarthritis (hip) | 30374069 | chr6:76164589 | A=0.11, C=0.89 |
| rs12209223 | Height | 30595370 | chr6:76164589 | A=0.11, C=0.89 |
| rs12209223 | Osteoarthritis (hip) | 30664745 | chr6:76164589 | A=0.11, C=0.89 |
| rs12209223 | Osteoarthritis (with total hip replacement) | 34450027 | chr6:76164589 | A=0.11, C=0.89 |
| rs12209223 | Hip circumference adjusted for BMI | 34021172 | chr6:76164589 | A=0.11, C=0.89 |
| rs12209223 | Height | 36224396 | chr6:76164589 | A=0.11, C=0.89 |
| rs12209223 | Height | 36224396 | chr6:76164589 | A=0.11, C=0.89 |
| rs12209223 | Height | 34594039 | chr6:76164589 | A=0.11, C=0.89 |
| rs12209223 | Height | 35831902 | chr6:76164589 | A=0.11, C=0.89 |
| rs12209223 | Hip circumference adjusted for BMI | 34021172 | chr6:76164589 | A=0.11, C=0.89 |
| rs12209223 | Body surface area | 36502284 | chr6:76164589 | A=0.11, C=0.89 |
| rs12209223 | Atrial fibrillation | 36653681 | chr6:76164589 | A=0.11, C=0.89 |
| rs12209223 | Multi-trait sex score | 37277458 | chr6:76164589 | A=0.11, C=0.89 |
| rs12209223 | Surgical hip osteoarthritis | 36376028 | chr6:76164589 | A=0.11, C=0.89 |
| rs12209223 | Non-surgical hip osteoarthritis | 36376028 | chr6:76164589 | A=0.11, C=0.89 |
| rs12209223 | Height | 38116116 | chr6:76164589 | A=0.11, C=0.89 |
| rs12209223 | Lung function (FEV1) | 36914875 | chr6:76164589 | A=0.11, C=0.89 |
| rs12209223 | Whole body fat free mass (UKB data field 23101) | 38538606 | chr6:76164589 | A=0.11, C=0.89 |
| rs12209223 | Height | 36224396 | chr6:76164589 | A=0.11, C=0.89 |
| rs12209223 | Height | 36224396 | chr6:76164589 | A=0.11, C=0.89 |
| rs17677724 | Total testosterone levels | 36653534 | chr5:128015370 | C=0.834, T=0.166 |
| rs1809889 | Height | 25282103 | chr12:124801226 | C=0.744, T=0.256 |
| rs1809889 | Hip circumference adjusted for BMI | 25673412 | chr12:124801226 | C=0.744, T=0.256 |
| rs1809889 | Osteoarthritis of the hip or knee (with total joint replacement) | 34450027 | chr12:124801226 | C=0.744, T=0.256 |
| rs1913707 | Osteoarthritis | 30664745 | chr4:13039440 | A=0.62, G=0.38 |
| rs1913707 | Osteoarthritis (hip) | 30664745 | chr4:13039440 | A=0.62, G=0.38 |
| rs1913707 | Osteoarthritis (with total hip replacement) | 34450027 | chr4:13039440 | A=0.62, G=0.38 |
| rs1913707 | Surgical hip osteoarthritis | 36376028 | chr4:13039440 | A=0.62, G=0.38 |
| rs1913707 | Non-surgical hip osteoarthritis | 36376028 | chr4:13039440 | A=0.62, G=0.38 |
| rs2268023 | Verbal learning | 35974141 | chr3:52819327 | A=0.431, T=0.569 |
| rs2416564 | Height | 30595370 | chr9:119370679 | C=0.422, T=0.578 |
| rs2416564 | Height | 36224396 | chr9:119370679 | C=0.422, T=0.578 |
| rs2521348 | Height | 36224396 | chr17:67499717 | C=0.601, T=0.399 |
| rs2605098 | Hip circumference variance | 31453325 | chr1:219643649 | A=0.335, G=0.665 |
| rs2605098 | Umbilical hernia | 34382107 | chr1:219643649 | A=0.335, G=0.665 |
| rs2605098 | Inguinal hernia | 34382107 | chr1:219643649 | A=0.335, G=0.665 |
| rs2605098 | Waist-to-hip ratio adjusted for BMI | 34021172 | chr1:219643649 | A=0.335, G=0.665 |
| rs2605098 | Hip index | 34021172 | chr1:219643649 | A=0.335, G=0.665 |
| rs2605098 | Hip circumference | 31453325 | chr1:219643649 | A=0.335, G=0.665 |
| rs2605098 | Waist-hip index | 34021172 | chr1:219643649 | A=0.335, G=0.665 |
| rs2605098 | Triglyceride levels in non-type 2 diabetes | 36269708 | chr1:219643649 | A=0.335, G=0.665 |
| rs2605098 | Sex hormone-binding globulin levels | 36653534 | chr1:219643649 | A=0.335, G=0.665 |
| rs28567725 | Triglycerides | 30275531 | chr16:53826028 | C=0.423, T=0.577 |
| rs28567725 | Addiction risk factors | 37250466 | chr16:53826028 | C=0.423, T=0.577 |
| rs2862851 | Hip minimal joint space width | 27701424 | chr2:70712802 | C=0.528, T=0.472 |
| rs3740129 | Osteoarthritis (with total hip replacement) | 34450027 | chr10:73767859 | A=0.431, G=0.569 |
| rs3740129 | Surgical hip osteoarthritis | 36376028 | chr10:73767859 | A=0.431, G=0.569 |
| rs4073717 | Height | 31562340 | chr5:170864021 | G=0.788, T=0.212 |
| rs4073717 | Fat-free mass | 30593698 | chr5:170864021 | G=0.788, T=0.212 |
| rs4073717 | Height | 30595370 | chr5:170864021 | G=0.788, T=0.212 |
| rs4073717 | Appendicular lean mass | 33097823 | chr5:170864021 | G=0.788, T=0.212 |
| rs4073717 | Fat-free mass | 30593698 | chr5:170864021 | G=0.788, T=0.212 |
| rs4073717 | Weight | 34594039 | chr5:170864021 | G=0.788, T=0.212 |
| rs4073717 | Height | 36224396 | chr5:170864021 | G=0.788, T=0.212 |
| rs4073717 | Height | 36224396 | chr5:170864021 | G=0.788, T=0.212 |
| rs4073717 | Hip circumference adjusted for BMI | 34021172 | chr5:170864021 | G=0.788, T=0.212 |
| rs4073717 | Body surface area | 36502284 | chr5:170864021 | G=0.788, T=0.212 |
| rs4073717 | Multi-trait sex score | 37277458 | chr5:170864021 | G=0.788, T=0.212 |
| rs4073717 | Multi-trait sex score | 37277458 | chr5:170864021 | G=0.788, T=0.212 |
| rs4073717 | Height | 38116116 | chr5:170864021 | G=0.788, T=0.212 |
| rs4073717 | Height | 36224396 | chr5:170864021 | G=0.788, T=0.212 |
| rs4073717 | Whole body fat free mass (UKB data field 23101) | 38538606 | chr5:170864021 | G=0.788, T=0.212 |
| rs4073717 | Height | 36224396 | chr5:170864021 | G=0.788, T=0.212 |
| rs4252548 | Lung function (FVC) | 30595370 | chr19:55879672 | C=0.979, T=0.021 |
| rs4252548 | Height | 30595370 | chr19:55879672 | C=0.979, T=0.021 |
| rs4252548 | Appendicular lean mass | 33097823 | chr19:55879672 | C=0.979, T=0.021 |
| rs4252548 | Appendicular lean mass | 33097823 | chr19:55879672 | C=0.979, T=0.021 |
| rs4252548 | Appendicular lean mass | 33097823 | chr19:55879672 | C=0.979, T=0.021 |
| rs4252548 | Osteoarthritis (hip) | 30374069 | chr19:55879672 | C=0.979, T=0.021 |
| rs4252548 | Osteoarthritis (hip) | 30664745 | chr19:55879672 | C=0.979, T=0.021 |
| rs4252548 | Osteoarthritis (with total hip replacement) | 34450027 | chr19:55879672 | C=0.979, T=0.021 |
| rs4252548 | Height | 36224396 | chr19:55879672 | C=0.979, T=0.021 |
| rs4252548 | Diffuse idiopathic skeletal hyperostosis flow score | 37156767 | chr19:55879672 | C=0.979, T=0.021 |
| rs4252548 | Surgical hip osteoarthritis | 36376028 | chr19:55879672 | C=0.979, T=0.021 |
| rs4252548 | Non-surgical hip osteoarthritis | 36376028 | chr19:55879672 | C=0.979, T=0.021 |
| rs4252548 | Height | 36224396 | chr19:55879672 | C=0.979, T=0.021 |
| rs4252548 | Whole body fat free mass (UKB data field 23101) | 38538606 | chr19:55879672 | C=0.979, T=0.021 |
| rs4252548 | Height | 36224396 | chr19:55879672 | C=0.979, T=0.021 |
| rs4411121 | Osteoarthritis (hip) | 34450027 | chr1:118757034 | C=0.655, T=0.345 |
| rs62578126 | Eyebrow thickness | 26926045 | chr9:129375338 | C=0.626, T=0.374 |
| rs62578126 | Appendicular lean mass | 33097823 | chr9:129375338 | C=0.626, T=0.374 |
| rs62578126 | Osteoarthritis (with total hip replacement) | 34450027 | chr9:129375338 | C=0.626, T=0.374 |
| rs62578126 | Medication use (antiglaucoma preparations and miotics) | 34594039 | chr9:129375338 | C=0.626, T=0.374 |
| rs62578126 | Alpha angle | 36662418 | chr9:129375338 | C=0.626, T=0.374 |
| rs62578126 | Glaucoma (primary open-angle) | 38382466 | chr9:129375338 | C=0.626, T=0.374 |
| rs62578126 | Glaucoma (primary open-angle) | 36777996 | chr9:129375338 | C=0.626, T=0.374 |
| rs66989638 | Height | 30595370 | chr2:106689736 | A=0.125, G=0.875 |
| rs66989638 | Osteoarthritis (with total hip replacement) | 34450027 | chr2:106689736 | A=0.125, G=0.875 |
| rs66989638 | Hip circumference adjusted for BMI | 34021172 | chr2:106689736 | A=0.125, G=0.875 |
| rs66989638 | Surgical hip osteoarthritis | 36376028 | chr2:106689736 | A=0.125, G=0.875 |
| rs67924081 | Type 2 diabetes | 34594039 | chr11:65342981 | A=0.744, G=0.256 |
| rs67924081 | Osteoarthritis (with total hip replacement) | 34450027 | chr11:65342981 | A=0.744, G=0.256 |
| rs67924081 | Latent-transforming growth factor beta-binding protein 3 levels | 34857772 | chr11:65342981 | A=0.744, G=0.256 |
| rs6855246 | Cortical surface area | 32193296 | chr4:103112470 | A=0.91, G=0.09 |
| rs6855246 | Insomnia | 30804565 | chr4:103112470 | A=0.91, G=0.09 |
| rs6855246 | General cognitive ability | 29844566 | chr4:103112470 | A=0.91, G=0.09 |
| rs6855246 | Brain region volumes | 31676860 | chr4:103112470 | A=0.91, G=0.09 |
| rs6855246 | Brain region volumes | 31676860 | chr4:103112470 | A=0.91, G=0.09 |
| rs6855246 | Brain region volumes | 31676860 | chr4:103112470 | A=0.91, G=0.09 |
| rs6855246 | Autism spectrum disorder or schizophrenia | 28540026 | chr4:103112470 | A=0.91, G=0.09 |
| rs6855246 | Intelligence (MTAG) | 29326435 | chr4:103112470 | A=0.91, G=0.09 |
| rs6855246 | vWF levels | 30586737 | chr4:103112470 | A=0.91, G=0.09 |
| rs6855246 | Attention deficit hyperactivity disorder or autism spectrum disorder or intelligence (pleiotropy) | 35764056 | chr4:103112470 | A=0.91, G=0.09 |
| rs6855246 | Inflammatory bowel disease or schizophrenia (pleiotropy) | 36753304 | chr4:103112470 | A=0.91, G=0.09 |
| rs6855246 | Occipital area (unadjusted for global measures) | 36893272 | chr4:103112470 | A=0.91, G=0.09 |
| rs6855246 | Short sleep duration (<5 hours) | 37770476 | chr4:103112470 | A=0.91, G=0.09 |
| rs79056043 | Osteoarthritis (hip) | 30664745 | chr12:59289598 | A=0.953, G=0.047 |
| rs79220007 | Hematocrit | 32888493 | chr6:26098474 | C=0.043, T=0.957 |
| rs79220007 | Hematocrit | 32888493 | chr6:26098474 | C=0.043, T=0.957 |
| rs79220007 | High light scatter reticulocyte count | 32888494 | chr6:26098474 | C=0.043, T=0.957 |
| rs79220007 | High light scatter reticulocyte percentage of red cells | 32888494 | chr6:26098474 | C=0.043, T=0.957 |
| rs79220007 | Apolipoprotein B levels | 32203549 | chr6:26098474 | C=0.043, T=0.957 |
| rs79220007 | LDL cholesterol levels | 32203549 | chr6:26098474 | C=0.043, T=0.957 |
| rs79220007 | High light scatter reticulocyte percentage of red cells | 27863252 | chr6:26098474 | C=0.043, T=0.957 |
| rs79220007 | High light scatter reticulocyte count | 27863252 | chr6:26098474 | C=0.043, T=0.957 |
| rs79220007 | Reticulocyte fraction of red cells | 27863252 | chr6:26098474 | C=0.043, T=0.957 |
| rs79220007 | Reticulocyte count | 27863252 | chr6:26098474 | C=0.043, T=0.957 |
| rs79220007 | Mean corpuscular hemoglobin concentration | 27863252 | chr6:26098474 | C=0.043, T=0.957 |
| rs79220007 | Red cell distribution width | 28957414 | chr6:26098474 | C=0.043, T=0.957 |
| rs79220007 | LDL cholesterol x physical activity interaction (2df test) | 30670697 | chr6:26098474 | C=0.043, T=0.957 |
| rs79220007 | Concentration of small LDL particles | 35213538 | chr6:26098474 | C=0.043, T=0.957 |
| rs79220007 | Cholesteryl ester levels in small LDL | 35213538 | chr6:26098474 | C=0.043, T=0.957 |
| rs79220007 | LDL cholesterol levels | 35213538 | chr6:26098474 | C=0.043, T=0.957 |
| rs79220007 | Alanine aminotransferase levels | 34594039 | chr6:26098474 | C=0.043, T=0.957 |
| rs79220007 | Platelet count | 34469753 | chr6:26098474 | C=0.043, T=0.957 |
| rs79220007 | Alanine aminotransferase levels | 35810165 | chr6:26098474 | C=0.043, T=0.957 |
| rs79220007 | Phospholipid levels in medium LDL | 35213538 | chr6:26098474 | C=0.043, T=0.957 |
| rs79220007 | Total lipid levels in medium LDL | 35213538 | chr6:26098474 | C=0.043, T=0.957 |
| rs79220007 | Concentration of medium LDL particles | 35213538 | chr6:26098474 | C=0.043, T=0.957 |
| rs79220007 | Total lipid levels in large LDL | 35213538 | chr6:26098474 | C=0.043, T=0.957 |
| rs79220007 | Total lipid levels in LDL | 35213538 | chr6:26098474 | C=0.043, T=0.957 |
| rs79220007 | Concentration of LDL particles | 35213538 | chr6:26098474 | C=0.043, T=0.957 |
| rs79220007 | Total cholesterol minus HDL-C levels | 35213538 | chr6:26098474 | C=0.043, T=0.957 |
| rs79220007 | Cholesteryl ester levels in large LDL | 35213538 | chr6:26098474 | C=0.043, T=0.957 |
| rs79220007 | Cholesterol levels in large LDL | 35213538 | chr6:26098474 | C=0.043, T=0.957 |
| rs79220007 | Cholesteryl ester levels in medium LDL | 35213538 | chr6:26098474 | C=0.043, T=0.957 |
| rs79220007 | Cholesteryl ester levels in LDL | 35213538 | chr6:26098474 | C=0.043, T=0.957 |
| rs79220007 | Cholesterol levels in medium LDL | 35213538 | chr6:26098474 | C=0.043, T=0.957 |
| rs79220007 | Osteoarthritis (hip) | 34450027 | chr6:26098474 | C=0.043, T=0.957 |
| rs79220007 | Triglycerides to total lipids ratio in large LDL | 38448586 | chr6:26098474 | C=0.043, T=0.957 |
| rs79220007 | Mean corpuscular hemoglobin concentration | 34594039 | chr6:26098474 | C=0.043, T=0.957 |
| rs79220007 | Delta of the red cell haemoglobins | 37596262 | chr6:26098474 | C=0.043, T=0.957 |
| rs79220007 | Surgical hip osteoarthritis | 36376028 | chr6:26098474 | C=0.043, T=0.957 |
| rs79220007 | Non-surgical hip osteoarthritis | 36376028 | chr6:26098474 | C=0.043, T=0.957 |
| rs79220007 | Serotransferrin levels | 34648354 | chr6:26098474 | C=0.043, T=0.957 |
| rs9475400 | Osteoarthritis (with total hip replacement) | 34450027 | chr6:55638258 | C=0.894, T=0.106 |
| rs9835230 | Osteoarthritis (hip) | 34450027 | chr3:189735461 | A=0.256, G=0.744 |

**Supplementary Table.2** Reports of SNPs across all phenotypes.
