# Supplementary figures and images for: Genetic insights into the risk of hip osteoarthritis on stroke: A single-variable and multivariable Mendelian randomization
Source: PLoS One. 2025 Jan 9;20(1):e0313032. doi: 10.1371/journal.pone.0313032 (PMC11717317; doi:10.1371/journal.pone.0313032)

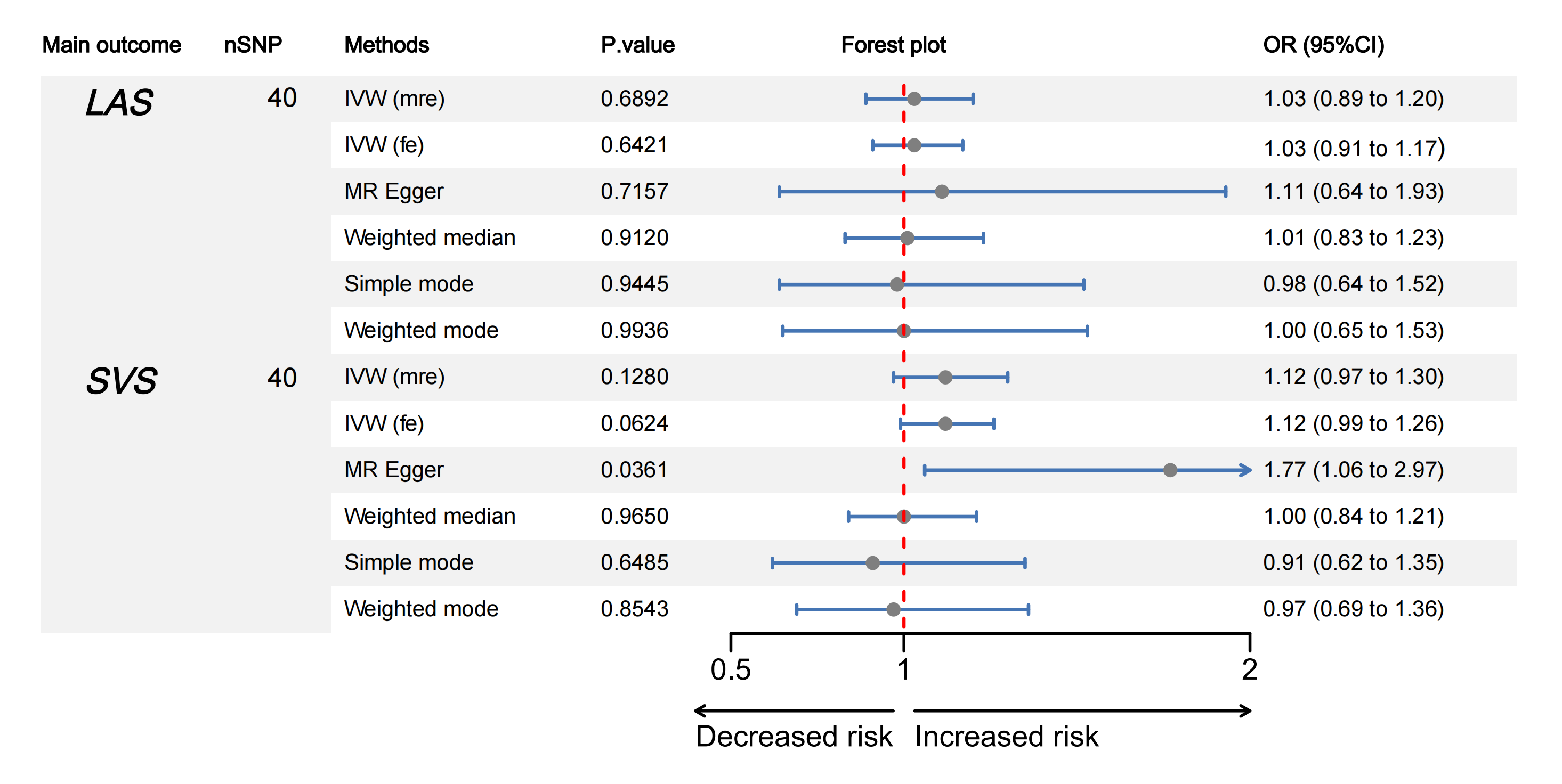

Supplement: S1 Fig — (TIF) [file pone.0313032.s003.tif]

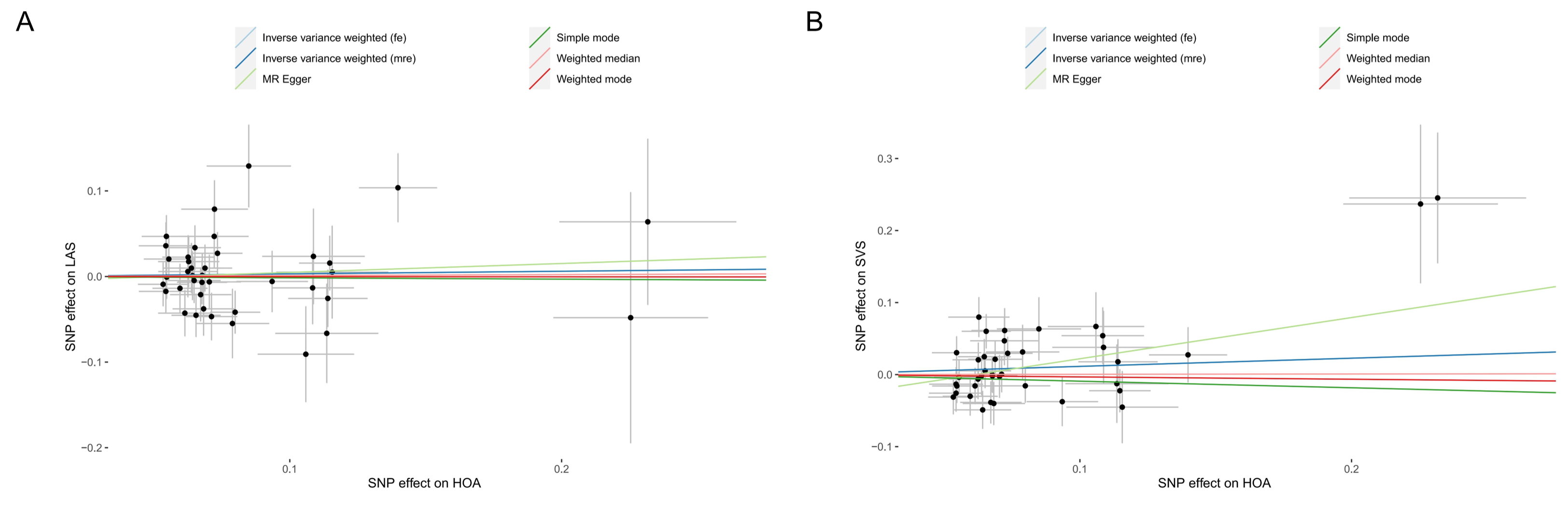

Supplement: S2 Fig — (TIF) [file pone.0313032.s004.tif]

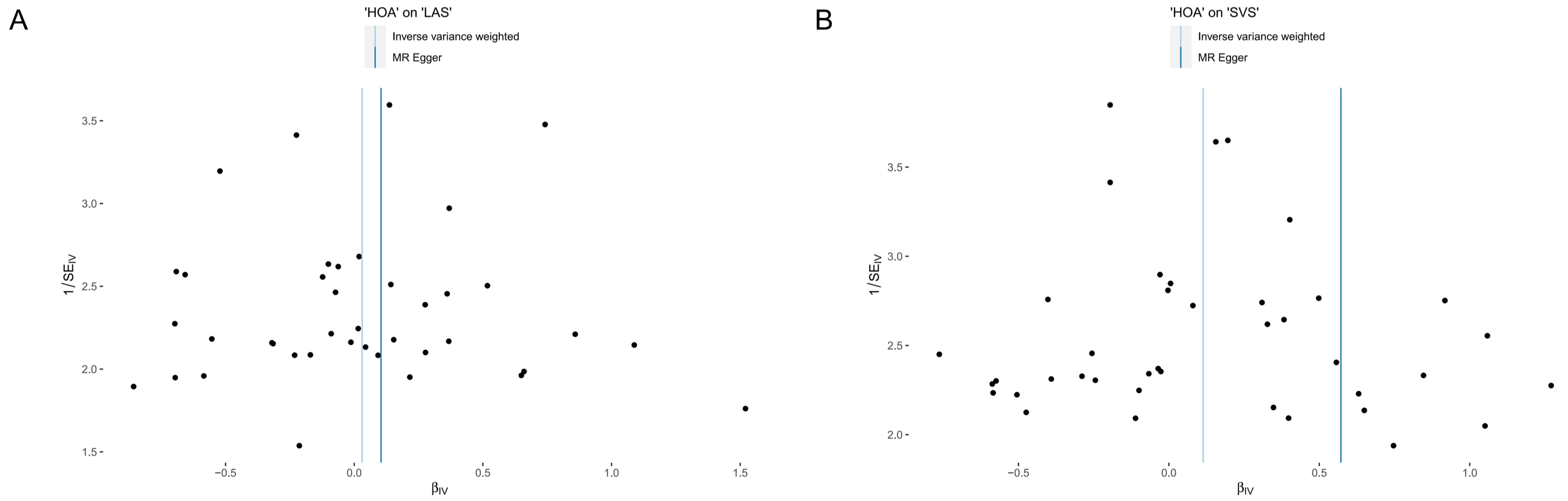

Supplement: S3 Fig — (TIF) [file pone.0313032.s005.tif]

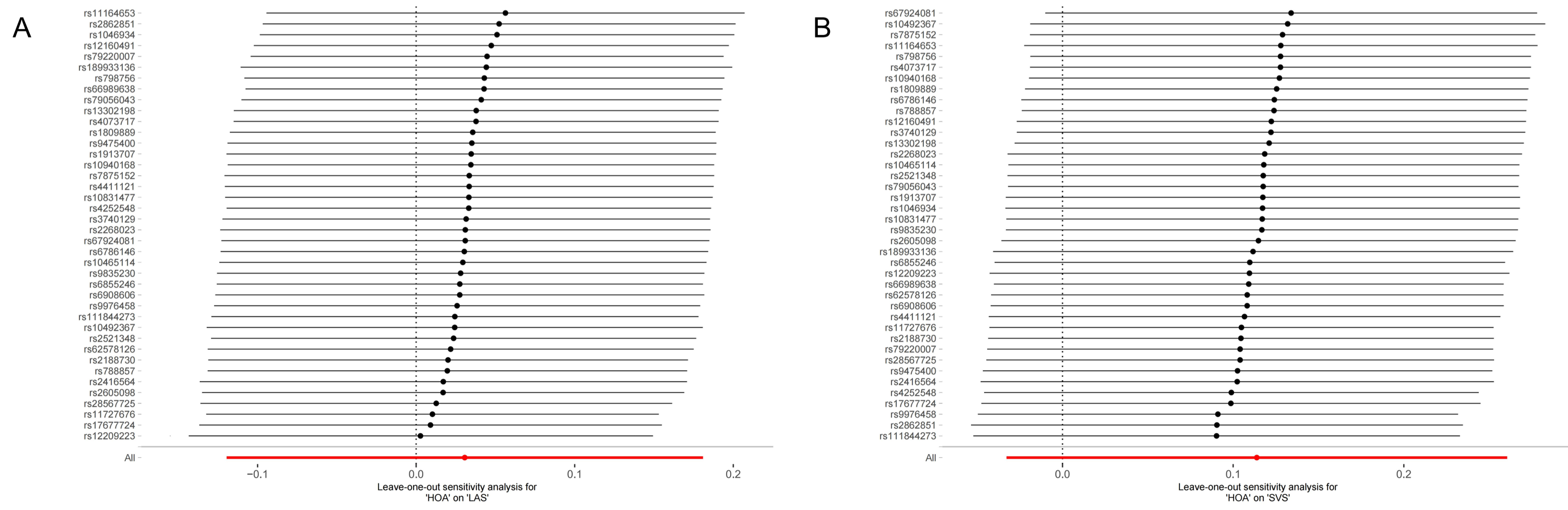

Supplement: S4 Fig — (TIF) [file pone.0313032.s006.tif]

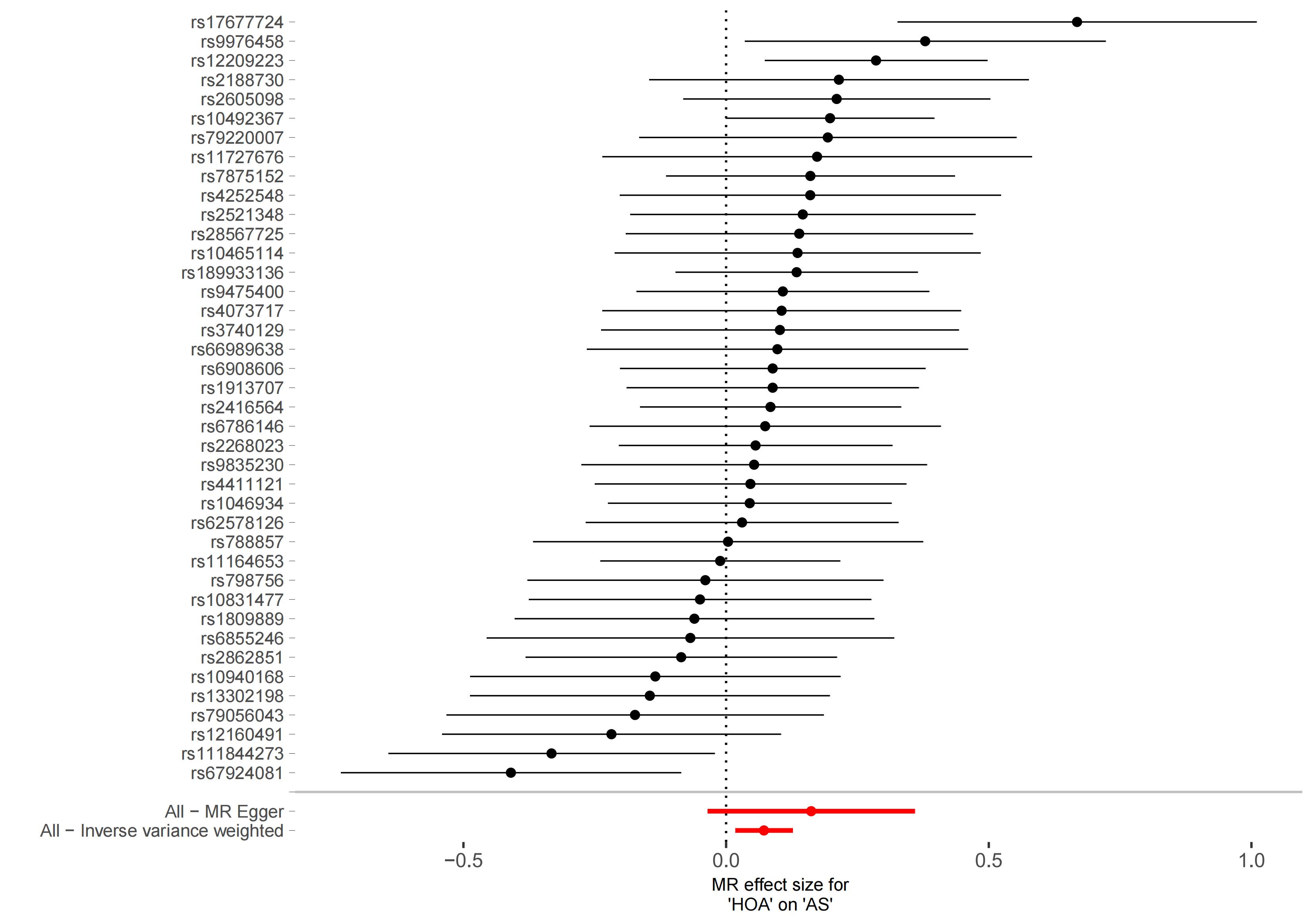

Supplement: S5 Fig — (TIF) [file pone.0313032.s007.tif]

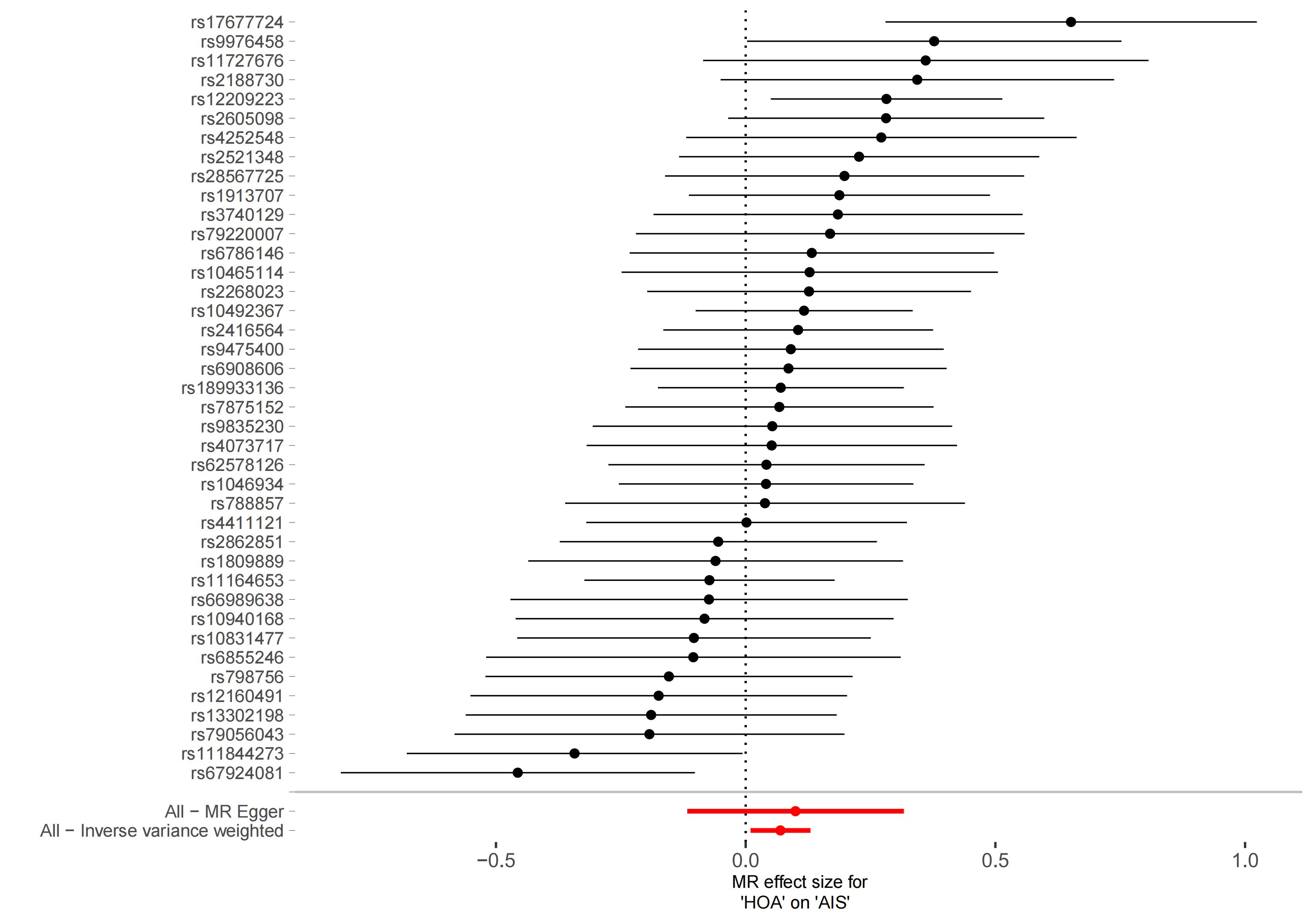

Supplement: S6 Fig — (TIF) [file pone.0313032.s008.tif]

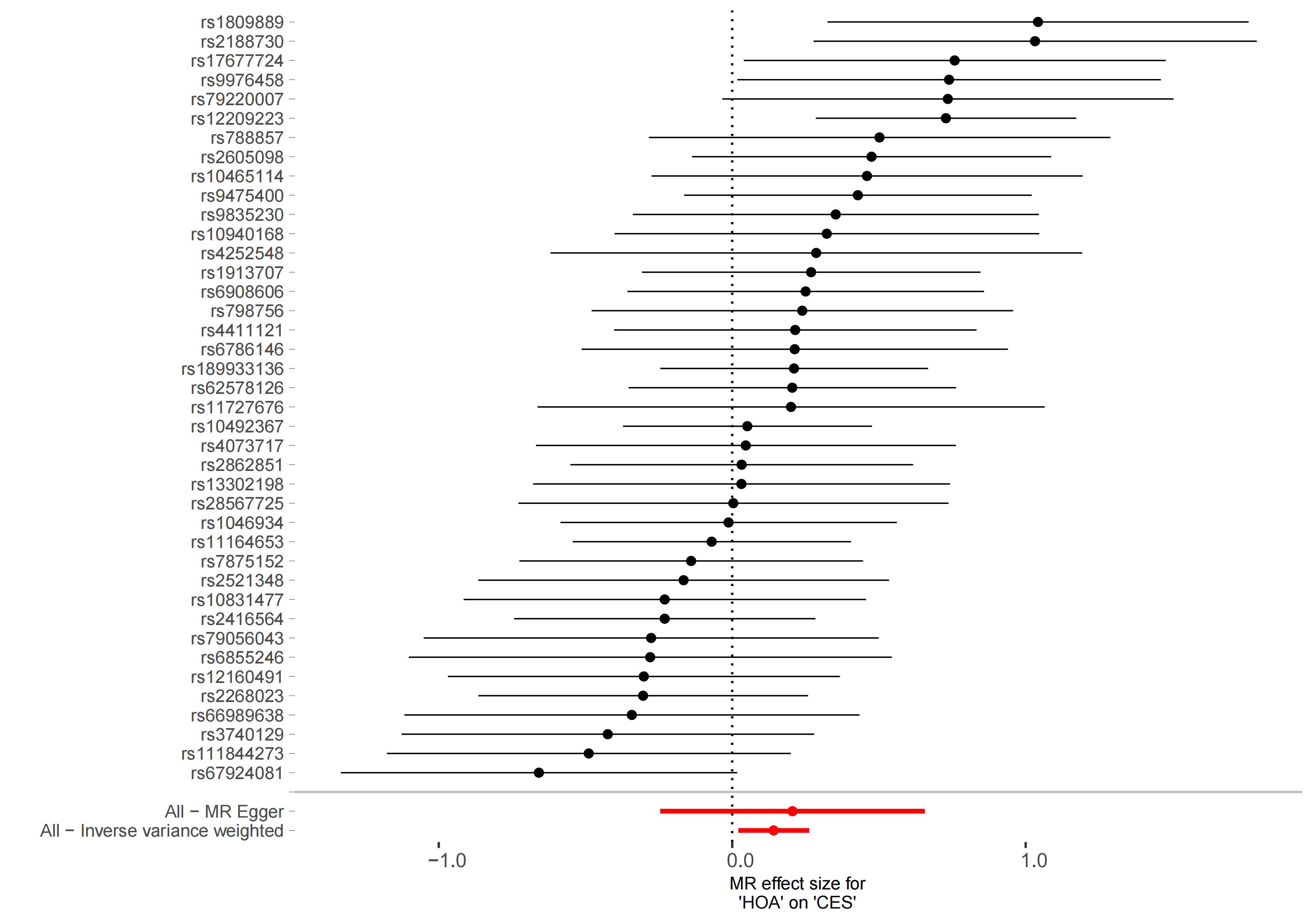

Supplement: S7 Fig — (TIF) [file pone.0313032.s009.tif]

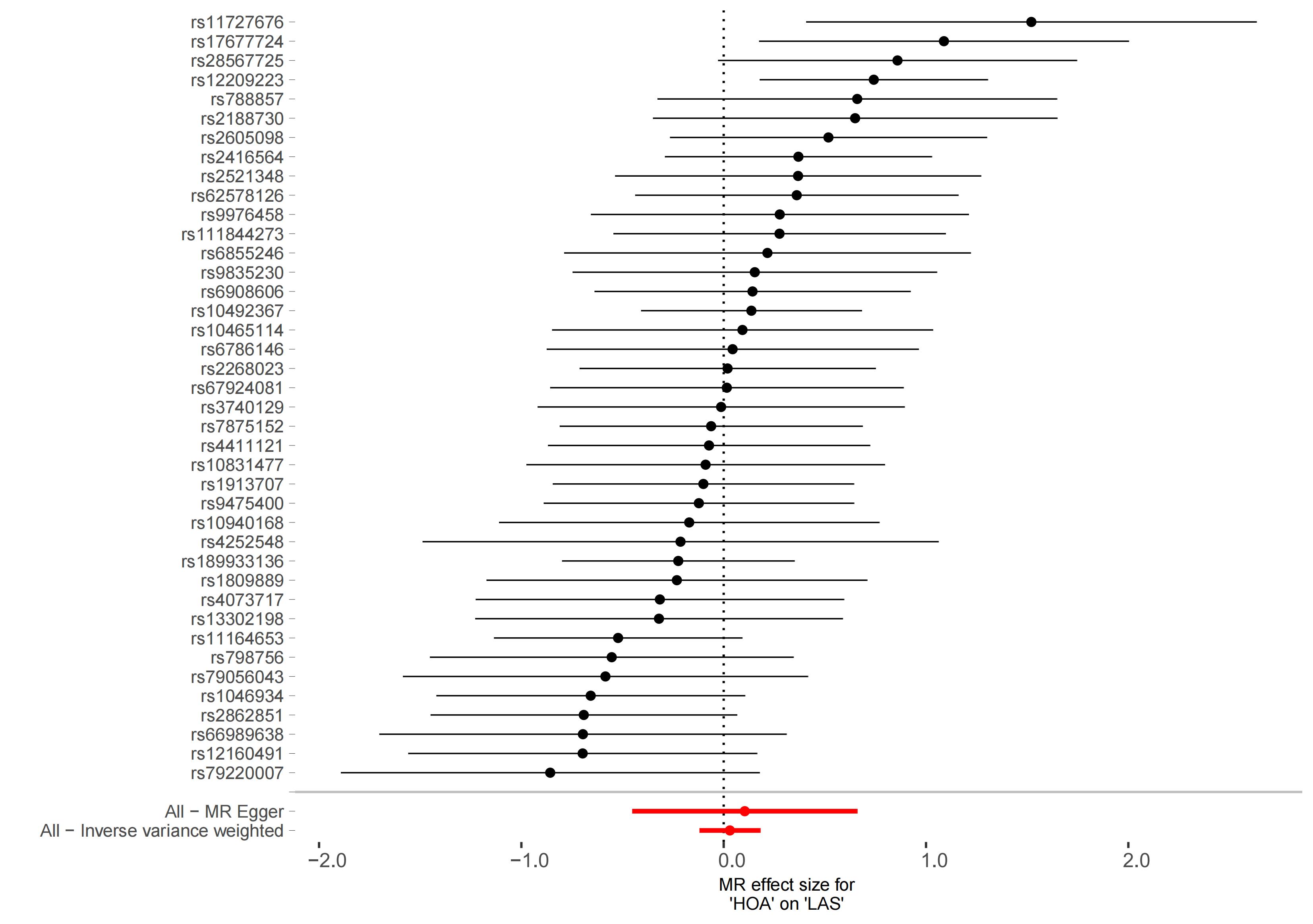

Supplement: S8 Fig — (TIF) [file pone.0313032.s010.tif]

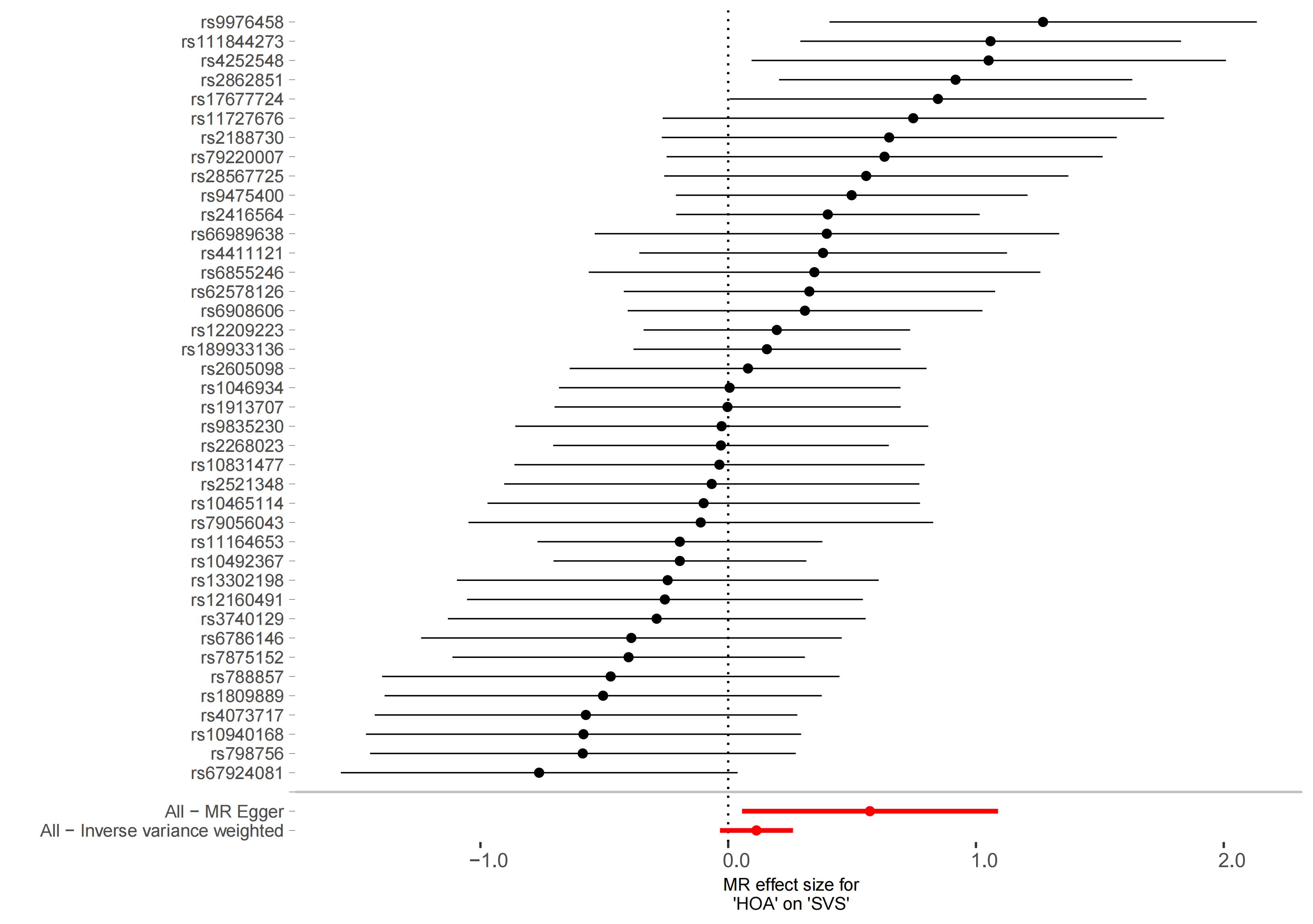

Supplement: S9 Fig — (TIF) [file pone.0313032.s011.tif]

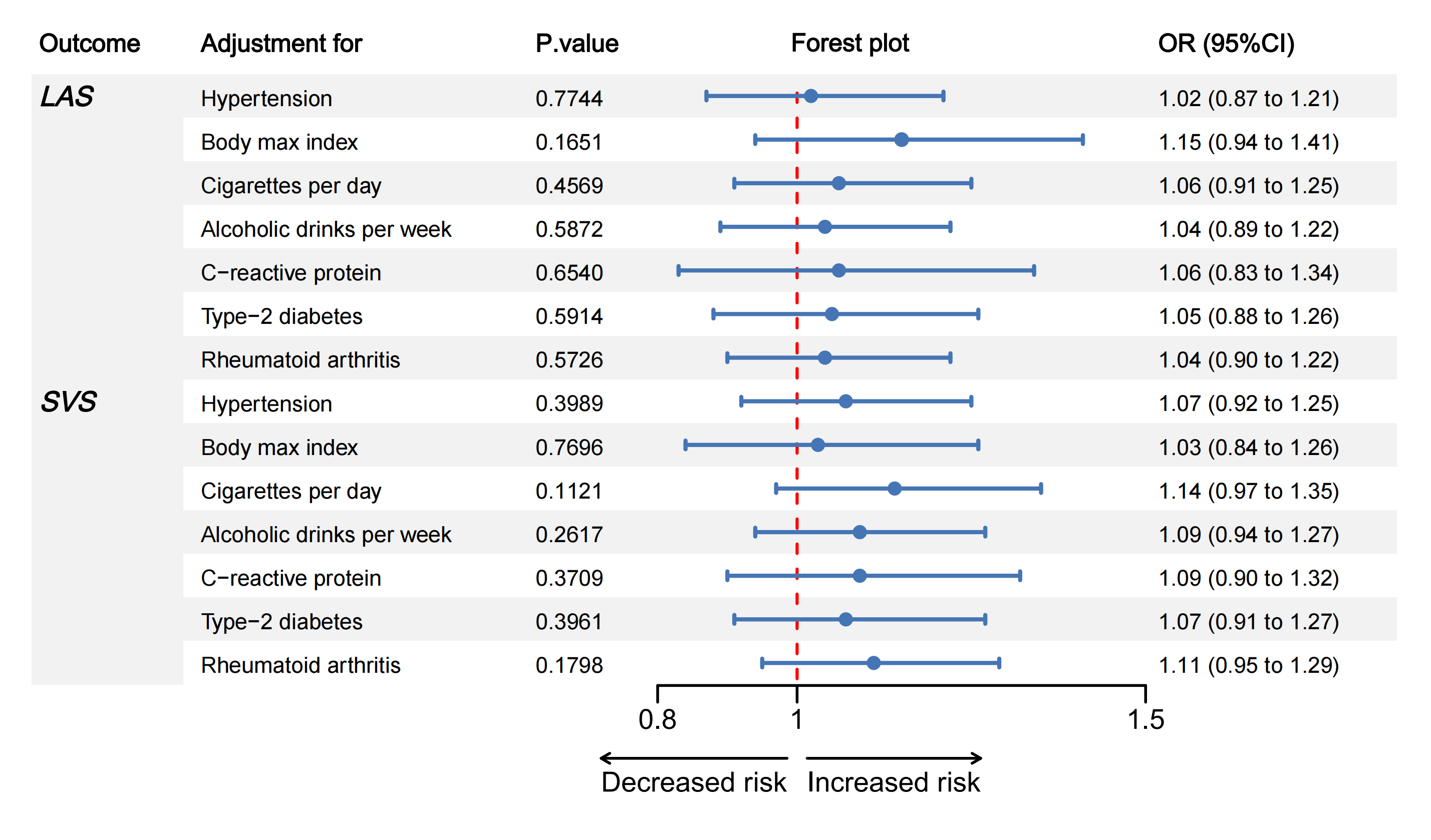

Supplement: S10 Fig — Hypertension, alcoholic drinks per week, cigarettes per day, T2D, CRP, BMI and rheumatoid arthritis were adjusted in the multivariate MR analyses. (TIF) [file pone.0313032.s012.tif]
